# The Burden of Dengue Fever and Chikungunya in Southern Coastal Ecuador: Epidemiology, Clinical Presentation, and Phylogenetics from the First Two Years of a Prospective Study

**DOI:** 10.4269/ajtmh.17-0762

**Published:** 2018-03-05

**Authors:** Anna M. Stewart-Ibarra, Sadie J. Ryan, Aileen Kenneson, Christine A. King, Mark Abbott, Arturo Barbachano-Guerrero, Efraín Beltrán-Ayala, Mercy J. Borbor-Cordova, Washington B. Cárdenas, Cinthya Cueva, Julia L. Finkelstein, Christina D. Lupone, Richard G. Jarman, Irina Maljkovic Berry, Saurabh Mehta, Mark Polhemus, Mercy Silva, Timothy P. Endy

**Affiliations:** 1 Center for Global Health and Translational Sciences, State University of New York (SUNY) Upstate Medical University, Syracuse, New York;; 2 Department of Medicine, State University of New York (SUNY) Upstate Medical University, Syracuse, New York;; 3 Department of Geography, University of Florida, Gainesville, Florida;; 4 Emerging Pathogens Institute, University of Florida, Gainesville, Florida;; 5 College of Life Sciences, University of Kwazulu-Natal, Durban, South Africa;; 6 Department of Microbiology and Immunology, State University of New York (SUNY) Upstate Medical University, Syracuse, New York;; 7 Department of Medicine, Universidad Técnica de Machala, Machala, El Oro, Ecuador;; 8 Laboratorio para Investigaciónes Biomédicas, Facultad de Ciencias de la Vida, Escuela Superior Politécnica del Litoral, Guayaquil, Guayas Province, Ecuador;; 9 Division of Nutritional Sciences, Cornell University, Ithaca, New York;; 10 Department of Public Health and Preventative Medicine, State University of New York (SUNY) Upstate Medical University, Syracuse, New York;; 11 Viral Diseases Branch, Walter Reed Army Institute of Research (WRAIR), Silver Spring, Maryland;; 12 Ministry of Health, Machala, El Oro, Ecuador

## Abstract

Here, we report the findings from the first 2 years (2014–2015) of an arbovirus surveillance study conducted in Machala, Ecuador, a dengue-endemic region. Patients with suspected dengue virus (DENV) infections (index cases, *N* = 324) were referred from five Ministry of Health clinical sites. A subset of DENV-positive index cases (*N* = 44) were selected, and individuals from the index household and four neighboring homes within 200 m were recruited (*N* = 400). Individuals who entered the study, other than the index cases, are referred to as associates. In 2014, 70.9% of index cases and 35.6% of associates had acute or recent DENV infections. In 2015, 28.3% of index cases and 12.8% of associates had acute or recent DENV infections. For every DENV infection captured by passive surveillance, we detected an additional three acute or recent DENV infections in associates. Of associates with acute DENV infections, 68% reported dengue-like symptoms, with the highest prevalence of symptomatic acute infections in children aged less than 10 years. The first chikungunya virus (CHIKV) infections were detected on epidemiological week 12 in 2015; 43.1% of index cases and 3.5% of associates had acute CHIKV infections. No Zika virus infections were detected. Phylogenetic analyses of isolates of DENV from 2014 revealed genetic relatedness and shared ancestry of DENV1, DENV2, and DENV4 genomes from Ecuador with those from Venezuela and Colombia, indicating the presence of viral flow between Ecuador and surrounding countries. Enhanced surveillance studies, such as this, provide high-resolution data on symptomatic and inapparent infections across the population.

## INTRODUCTION

The region of the Americas is facing an unprecedented public health crisis of co-occurring epidemics of illness due to dengue virus (DENV), chikungunya virus (CHIKV), and Zika virus (ZIKV). These arboviruses cause acute febrile illness and are transmitted to humans by the female *Aedes aegypti* and *Aedes albopictus* mosquitoes.

Dengue fever is caused by an infection by one of the serotypes of the mosquito-borne DENV (DENV 1–4, family Flaviviridae, genus *Flavivirus*). Clinical manifestations range from mild illness (i.e., fever, rash, and joint pain) to severe illness characterized by pathologic vascular permeability leading to hemorrhage, shock, and sometimes death.^1^ Over the last three decades, the distribution, severity, and incidence of DENV have increased in Latin America, from 16.4 cases per 100,000 in the 1980s to 71.5 cases per 100,000 from 2000 to 2007.^2,3^ Current estimates of apparent DENV infection in the Americas range from 1.5 million^4^ to 13.3 million^5^ infections per year. In 2015, 2.35 million DENV infections were reported in the Americas, leading to 10,200 severe infections and 1,181 deaths.^6^

More recently, CHIKV and ZIKV have emerged and caused major epidemics in the same populations in the Americas. The first CHIKV infections (family Togaviridae, genus *Alphavirus*) were reported in the Americas in 2013, resulting in more than 2.5 million suspected and confirmed cases to date.^7^ The first ZIKV infections (family Flaviviridae, genus *Flavivirus*) were reported in Brazil in 2015.^8,9^ To date, 806,898 suspected and confirmed cases of ZIKV have been reported from the Americas (as of January 4, 2018).^10^

In Ecuador, DENV causes the greatest burden of mosquito-borne febrile illness. In 2014 and 2015, the years of this study, 16,908 and 44,104 cases per year, respectively, were reported.^11^ Historically, DENV was eliminated from Ecuador in the 1950s through the use of dichlorodiphenyltrichloroethane and other measures to control *Ae. aegypti*, the primary vector in Ecuador.^12,13^ Following the weakening of the vector control program and the reinvasion of *Ae. aegypti* in the 1970s and 1980s, DENV1 reemerged in Ecuador in 1988 and caused a major epidemic of classic dengue fever.^14^ From 1993 to 1999, three serotypes circulated: DENV1, DENV2 (American strain), and DENV4. In 2000, DENV3 and DENV2 (Asian strain) were identified and the first cases of severe hemorrhagic dengue were subsequently reported.^15^

Today, the burden of DENV is greatest in the coastal lowland region of Ecuador, the site of the present study. Prior studies in southern coastal Ecuador indicate that DENV transmission is highly seasonal, with the greatest incidence of disease and density of mosquito vectors from February to May, the hot and rainy season, and lower transmission throughout the rest of the year.^16,17^ Dengue virus epidemics in the region are associated with El Niño climate events that result in warmer air temperatures.^16^ Local social–ecological risk factors for DENV infections and *Ae. aegypti* proliferation in this region include adjacent abandoned properties, interruptions in piped water, shaded patios, lack of use of mosquito bed nets, lack of fumigation inside the home, poor housing conditions, inadequate piped water, gaps in knowledge about DENV transmission, and water storage habits.^17–20^

The first autochthonous CHIKV infections were reported in Ecuador at the end of 2014; to date, 35,893 suspected and confirmed cases have been reported (as of December 22, 2017).^7^ The first autochthonous ZIKV infections were confirmed in Ecuador on January 7, 2016. A total of 6,351 suspected and confirmed cases of ZIKV infections have been reported (as of January 4, 2018), including 14 cases of congenital syndrome associated with ZIKV infections, which were first reported in May 2017.^10^

In Ecuador, suspected and confirmed DENV, CHIKV, and ZIKV cases require mandatory notification to the Ministry of Health (MoH). The MoH in Ecuador follows the 2009 World Health Organization dengue diagnostic guidelines.^1^ The national surveillance system is based on passive surveillance of cases from MoH clinics and hospitals. A subset of suspected cases are confirmed for DENV using nonstructural protein 1 (NS1) antigen and immunoglobulin M (IgM) enzyme-linked immunosorbent assays (**ELISA**) in local diagnostic laboratories operated by the MoH. A subset of cases are confirmed for DENV, CHIKV, and ZIKV using quantitative polymerase chain reaction (PCR) at the national reference laboratory of the National Institute for Public Health Research of the MoH. Suspected infections trigger focal vector control interventions in the infected home and surrounding homes by the MoH (i.e., fogging, indoor residual spraying, source reduction, and larvicide application).

There have been prior enhanced surveillance studies to estimate the burden of dengue fever in Asia^21–24^ and Latin America,^25–31^ with study designs ranging from pediatric to adult cohorts, tracking of school-based absentees, use of sentinel clinics, and community-based cluster investigations. In general, these studies found that enhanced surveillance methods identified a greater number of DENV infections, especially mild and inapparent infections, compared with traditional passive surveillance systems. Enhanced surveillance studies generate high-resolution data on the spatiotemporal distribution of symptomatic and inapparent infections across the population. This is especially important in settings and in subgroups with low health-care–seeking behavior or limited access to health centers. These data allow the public health sector to more accurately estimate the social and economic burden of the disease, allowing for more informed decision-making regarding the allocation of scarce resources. These studies can also inform the design and implementation of interventions targeted at high-risk groups, such as vaccination campaigns or vaccine trials.

Here, we present the results of the first 2 years of an active surveillance study in Ecuador. The aim of this study was to characterize the epidemiology, clinical presentation, and viral phylogenetics of DENV. We also present the epidemiology and clinical characteristics of CHIKV during the first CHIKV outbreak. This study is a part of a long-term partnership with the MoH of Ecuador focused on strengthening febrile vector-borne disease surveillance in southern coastal Ecuador, providing high-resolution epidemiological information for the region.^32^

## MATERIALS AND METHODS

### Definitions.

*Index cases* are hospitalized patients and outpatients with a clinical diagnosis of an acute DENV infection who enrolled in the study. *Initiate index cases* are index cases that tested positive for DENV and were randomly selected to initiate a cluster investigation. *Associates* are study subjects who resided in the home of the initiate index case and/or in the four neighboring homes located in the cardinal directions at a maximum distance of 200 m from the initiate index household. The four associate homes plus the initiate index case home are referred to as a *cluster*.

Study subjects were considered to have an *acute DENV infection* if they tested positive by NS1 rapid test (RT), NS1 ELISA, or reverse transcription polymerase chain reaction (RT-PCR). If the subjects were negative for those three tests, but were positive by IgM ELISA, they were classified as having a *recent DENV infection*. Individuals were classified as *uninfected with DENV* if they were negative for NS1 RT, NS1 ELISA, RT-PCR, and IgM ELISA. Individuals who tested negative for all of the tests except for the presence of immunoglobulin G (IgG) antibodies were not classified. Individuals who tested positive for CHIKV or ZIKV by RT-PCR were classified as having an *acute CHIKV* or *acute ZIKV infection*.

We define a *symptomatic* individual as an associate with one or more dengue-like symptoms. By definition, all index cases are symptomatic. Prior studies that report symptomatic illness defined symptomatic as febrile,^24,33^ whereas we use a broader definition of symptomatic to include any dengue-like symptom (e.g., headache, muscle/joint pain, retro-orbital pain, abdominal pain, drowsiness/lethargy, fever, and rash) because symptoms other than fever were more frequently reported by associates with acute DENV infections (Supplemental Table 1). An *inapparent* infection is defined as an infection in an associate who has no dengue-like symptoms.

### Ethics statement.

This study protocol was reviewed and approved by Institutional Review Boards at the State University of New York (SUNY) Upstate Medical University, Cornell University, the Human Research Protection Office of the U.S. Department of Defense, the Luis Vernaza Hospital in Guayaquil, Ecuador, and the Ecuadorean MoH. Before the start of the study, all participants engaged in a written informed consent or assent process, as applicable. If the participant was unable to participate in the consent or assent process, an adult representative documented their consent. Children aged 7–17 years signed an assent statement and parents signed an informed consent. Parents signed an informed consent on behalf of children aged greater than 6 months to less than 7 years. The study included children (> 6 months) to adults (index cases) who were evaluated in sentinel clinics or the hospital with a clinical diagnosis of acute DENV infection. Before signing the informed consent, index cases were informed that they might be randomly selected to participate in a cluster investigation (initiate index cases). Additional study subjects include associate children (> 6 months) and adults, who resided in the cluster homes.

### Study site.

**Figure 1.**
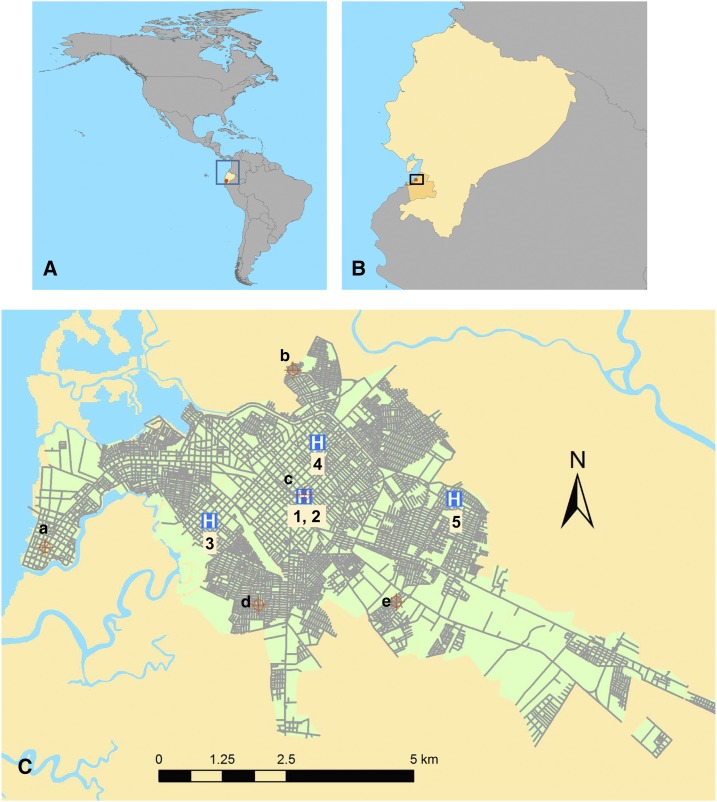
Map of the study site: (**A**) location of Ecuador in the Americas. (**B**) location of El Oro province in Ecuador, the city of Machala indicated as a red dot. (**C**) The city of Machala, showing the five Ministry of Health clinical sites/hospital: 1) Mabel Estupiñan Clinic, 2) Teofilo Davila Hospital, 3) Brisas del Mar Clinic, 4) El Paraiso Clinic, 5) Rayito de Luz Clinic. The locations of meteorological stations are indicated by (a**–**e) as follows: (a) Puerto Bolivar; (b) Los Esteros; (c) Mabel Estupiñan; (d) Florida; (e) Crucitas. This figure appears in color at www.ajtmh.org.

Machala, Ecuador (population 280,694, capital of El Oro province), is a port city located along the Pan American Highway, near the Ecuador–Peru border (Figure 1). Machala has among the highest incidence rates of DENV in Ecuador and exceptionally high *Ae. aegypti* densities compared with other countries in Latin America and Asia.^17,34,35^ In 2014 and 2015, 1,196 and 2,791 DENV cases, respectively, were reported from Machala (annual incidence of 42.6 cases per 10,000 people in 2014 and 99.4 cases per 10,000 people in 2015).^36^ The first local cases of CHIKV were reported by the MoH in May 2015 and the first cases of ZIKV were reported in February 2016. Machala is a strategic location to monitor and investigate DENV—and now CHIKV and ZIKV—transmission dynamics because of its location near an international border and port, and the historically high incidence of mosquito-borne diseases.

Sentinel clinical sites operated by the MoH in Machala were selected based on historically reported DENV cases and the resources that they were able to offer for coordinating and supporting the methods of this surveillance study. Of the 23 MoH clinics in Machala, four were selected. These included the clinics Brisas del Mar, Rayito de Luz, Mabel Estupiñan, and El Paraiso. In addition, the Teófilo Dávila Hospital of the MoH was included because it is the principal public hospital of the province, where the MoH clinics refer patients with severe DENV infections.

### Passive and active surveillance study design.

Hospitalized patients and outpatients with a clinical diagnosis of an acute DENV infection (index cases), as determined by MoH physicians, were referred to our study technician or nurse and were invited to participate in the study. Consent was obtained and the following data were collected using a customized database on an Ipad (FileMaker Pro Advanced 13.0v5; FileMaker Inc., Santa Clara, CA): patient demographics, home address, primary reason for seeking medical care, date of onset of fever, symptoms within the last 7 days, medications, and aural temperature. Data were uploaded daily and stored in a secure cloud-based server (GoZync). At the time of clinical evaluation, a 20-mL blood specimen (adjusted for age and weight by the United States National Institutes of Health criteria) was obtained by venipuncture from each participant. The samples were processed at our diagnostic laboratory at the hospital. Serum samples were used to test for acute DENV infections using NS1 RT (PanBio Dengue Early Rapid Test). Nonstructural protein 1 tests were run the same day that the index case was recruited into the study. Additional serum, cells, and plasma were separated via centrifugation and aliquoted in multiple tubes and stored at −80°C.

Each week, up to four index cases that were positive for DENV infection were randomly selected to be initiate index cases, and they were invited to participate in the active surveillance component of this study. The study team visited the household of the initiate index case and the nearest neighboring homes in each of the four cardinal directions, at a distance of less than 200 m from the index household, the typical flight range of the *Ae. aegypti* mosquito. All household members (associates) from this cluster of homes were invited to participate in the study. Investigations in clusters began within 2 days of the initiate index case entering the study. The diagnostic tests and clinical assessments described previously for index cases were repeated for all associates. The location (latitude and longitude) of each home was recorded using handheld Garmin global positioning system units. Passive and active surveillance study designs were optimized in a prior study by the Armed Forces Research Institute of Medical Sciences (AFRIMS) in Kamphaeng Phet Province, Thailand.^24^

### Diagnostic assays.

Additional diagnostic testing for DENV was conducted using serum samples and commercial ELISA kits (Panbio) to test for NS1 (Dengue Early ELISA), IgM (Dengue Capture IgM), and IgG (Dengue Capture IgG). We classified participants as having a primary DENV infection if the ratio of IgM to IgG was ≥ 1.8 and a secondary DENV infection if the ratio was less than 1.8.^24,37,38^

Specimens were shipped to SUNY Upstate Medical University for testing by qualitative RT-PCR assays for DENV1–4, CHIKV, and ZIKV. All samples from 2014 and 2015 were screened for DENV1–4. Samples from index cases in 2014 and index cases and associates in 2015 were screened for CHIKV. Only samples from index cases and associates in 2015 were screened for ZIKV. All analyses were performed on a BioRad DNA Engine Chromo 4 System with MJ Opticon Monitor Analysis Software. For DENV1–4 analysis, total RNA was extracted from 140 μL of human serum specimens using the QIAamp^®^ Viral RNA Mini Kit (Catalog number 52906; Qiagen, Germantown, MD) according to the manufacturer’s suggested protocol and resuspended in 50 μL of buffer. Ten microliters of RNA (or the equivalent of 28 μL of serum) was used in a 20 μL reverse transcriptase reaction, of which 5 μL of the resulting cDNA was used for PCR. All samples and controls were analyzed in duplicate in a multiplex RT-PCR for 45 cycles using SuperScript III Platinum One-Step qRT-PCR System (Catalog number 11732-020; Life Technologies^™^, Grand Island, NY) based on the United States Centers for Disease Control and Prevention (CDC) DENV1–4 Real Time RT-PCR Assay (Catalog number KK0128; CDC)^39^ and a published assay.^40^ Samples were classified as positive according to a suggested *C*(*t*) value of ≤ 37.0, which coincides with a cutoff based on CDC recommendations for identifying positive DENV samples.^39^ For ZIKV and CHIKV analyses, total RNA was extracted from human serum specimens using the QIAamp Viral RNA Mini Kit (Catalog number 52906; Qiagen) according to a modified assay developed at the Walter Reed Army Institute of Research (WRAIR), Viral Diseases Branch. All samples and controls were analyzed in duplicate in a multiplex RT-PCR using TAQMAN Fast Virus 1-Step Mix (Catalog number 4444432; Life Technologies). The CHIKV primer and probe set (hexachloro-fluorescein reporter) was adapted from an AFRIMS protocol, Set 3, which was designed specifically for the Asian genotype CHIKV strain presently in the Caribbean and verified using Synthetic CHIKV RNA control (Catalog number VR-3246SD; American Type Culture Collection, Manassas, VA). The ZIKV primer and probe set (fluorescein amidite reporter) was based on the AFRIMS protocol that was adapted from a published assay^41^ and verified using RNA extracted from ZIKV culture fluid (Catalog number 0810092CF; ZeptoMetrix Corp., Buffalo, NY). Both primer and probe sets were specific for their respective viral target and did not detect other viruses (DENV1–4, yellow fever virus, and Japanese encephalitis virus). Samples were classified as positive based on the same cutoff value used for DENV (*C*[*t*] value of ≤ 37.0). Primers and probes for DENV, CHIKV, and ZIKV are shown in Supplemental Tables 2 and 3.

### Statistical analysis.

Statistical analyses were conducted using R (version 3.3.3) in RStudio (version 1.0.136), using the “base” and “psych” packages for summary statistics. Student’s *t* test was used to determine differences in continuous variables and χ^2^ or Fisher’s exact test were used for proportions.

### Sequencing and consensus assembly.

Samples from 2014 that were DENV positive by RT-PCR were sent to WRAIR, Viral Diseases Branch, for full-length sequencing. The samples were extracted using a Qiagen QIAamp viral mini RNA extraction kit in accordance with the manufacturer’s protocols. Full genome was amplified on Fluidigm Access Array system using DENV serotype–specific primers and the Life Technologies III One-Step RT-PCR system with Platnimum^®^ Taq High Fidelity polymerase, followed by cDNA quality check using Agilent Bioanalyzer DNA7500 kit and RT-PCR product purification. Purified RT-PCR products were quantified using the Invitrogen Quant-iT^™^ PicoGreen dsDNA Reagent and Kit following the manufacturer’s protocols. MiSeq library preparation included dilution of purified amplicon products to 0.2 ng/μL, tagmentation using 5 μL of each dilution stock as input DNA, neutralization of each Nextera^®^ XT Tagmentation (llumina Inc, San Diego, CA) reaction using 5 μL neutralize tagment buffer, PCR amplification using index primers from Nextera XT Index kit version 2 set C, PCR clean-up using 25 μL per reaction of Beckman Counter AMPure XP beads, and library normalization using applicable reagents provided in the Nextera XT DNA Library Preparation kit. After normalization, each library was pooled and sequenced using the Illumina MiSeq reagent kit (version 2, 500 cycles) and Illumina MiSeq next generation sequencer in accordance with Illumina protocols.

Construction of consensus genomes was performed using ngs_mapper v1.2.4 in-house–developed pipeline (available on github, http://github.com/VBDWRAIR/ngs_mapper). Briefly, raw FASTQ data were stripped of barcodes and adapters and subjected to read filtering using a quality threshold of Q25. Remaining reads were further end-trimmed using a quality threshold of Q25 using Trimmomatic.^42^ Trimmed reads with quality > Q25 were initially mapped to a set of reference sequences to determine the best reference fit for each of the samples. Following reference determination, reads from each of the samples were remapped to their closest related reference genome to maximize the number of mapped reads. Reference mapping was performed using the Burrows-Wheeler Aligner-Maximal Exact Matches algorithm.^43^ Assemblies were further processed using samtools version 0.1^44^ and an in-house–developed python program called *basecaller.py* to produce an adapted variant call format for each segment, in parallel, which incorporates genomic ambiguity inherent in RNA viruses into the final consensus genome for that sample based on thresholds set by the investigator. Threshold for consensus genomic reconstruction for ambiguity incorporation was set at 20% for this analysis, meaning if any site contained a different nucleotide call that was present at 20% or greater in the dataset (taking quality of call into account), the site was given an ambiguous base call (according to International Union of Pure and Applied Chemistry conventions). Consensus sequences for all samples were constructed, in parallel, from the adapted VCF output. All consensus sequences were further manually quality checked. Statistics and graphics illustrating read depth and quality of mappings for each sample across each segment produced by the pipeline were carried out using matplotlib.^45^

### Phylogenetic analyses.

The five sequenced full-genome DENV1 samples were aligned to a set of full-genome DENV1 reference sequences obtained from GenBank using MEGAv6.^46^ The 131 reference genomes were selected to represent 1) all DENV1 genotype lineages, for accurate genotype determination, 2) wide sampling time periods, with a focus on the most recently sampled genomes (2009–2016), 3) most geographical regions, with a focus on Central and South America. In addition, the top 20 genomes matching the five genomes from Ecuador through Basic Local Alignment Search Tool (BLAST)^47^ were added to the reference dataset. A set of 140 full-genome DENV2 reference sequences was obtained from GenBank following the same criteria as for DENV1 and aligned to the 27 DENV2-sequenced genomes from Ecuador. Likewise, a set of 100 full-genome DENV4 reference sequences was obtained from GenBank following the same criteria as for DENV1, and aligned to the single DENV4-sequenced genome from Ecuador. We were unable to sequence DENV3 because of the limited sample volume. Genetic sequences are deposited in GenBank under accession numbers KY474303–KY474335.

We determined the best-fit models of evolution for DENV1, DENV2, and DENV4 datasets using jModelTest v2.1.7 with Akaike information criterion (AIC) and Bayesian information criterion (BIC).^48^ Maximum likelihood (ML) phylogenetic trees for DENV1, DENV2, and DENV4 datasets were inferred using Phyml v 4.9.1.^49,50^ The model of evolution used for the full-genome tree inferences was GTR + *I* + *Γ* (general time reversible with empirically estimated proportion of invariant sites and gamma distribution of among-site variation, four categories), for all three DENV serotypes. The tree space was searched heuristically using the best of Nearest Neighbor Interchanges and Subtree Pruning and Regrafting. Node confidence values were determined by approximate likelihood ratio test (aLRT) using the nonparametric Shimodaira–Hasegawa approach. Node confidence values of > 0.75 are considered good support. The resulting trees were rooted by the KR919820 sylvatic reference genome^51^ for DENV1 and by the sylvatic genotype out-groups for DENV2 and DENV4.

## RESULTS

**Figure 2.**
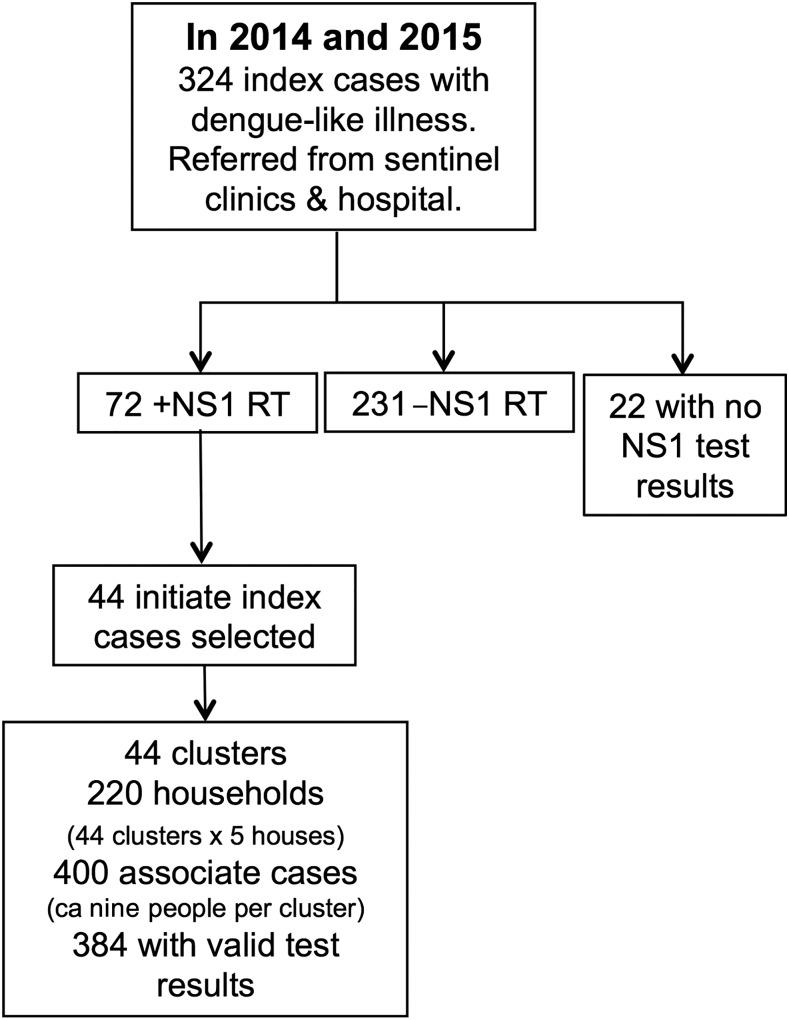
Dengue virus surveillance study design in Machala, Ecuador. NS1 RT = nonstructural protein 1 rapid test.

From January 1, 2014 through December 31, 2015, we recruited 324 index cases with suspected DENV infections from the five clinical sites in Machala, Ecuador (Figures 1 and 2). A subset of 310 index cases (186 in 2014 and 124 in 2015) had valid test results and were included in this study (Table 1). A total of 72 index cases were positive by NS1 RT, and from these, we randomly selected 44 initiate index cases, from which 400 associates were recruited into the study. A subset of 384 associates (298 in 2014, 86 in 2015) had valid test results and were included in this study.

**Table 1:** Demographic data and infection status of index cases and associates

	2014		2015	
	Index cases	Associates	Index cases	Associates
	*N* = 186	*N* = 298	*N* = 124	*N* = 86
Age in years, mean (SD)	20.6 (15.5)	35.3 (19.1)	28.0 (18.6)	38.8 (20.0)
Gender, % female	90/186 (48.4%)	195/295 (66.1%)	68/124 (54.8%)	58/86 (67.4%)
Temperature > 38°C	30/185 (16.2%)	2/290 (0.7%)	23/124 (18.5%)	0/86 (0%)
Fever in the prior 7 days	179/185 (96.8%)	33/285 (11.6%)	119/124 (96.0%)	3/83 (3.6%)
DENV infection				
Acute infection	75/186 (40.3%)	45/298 (15.1%)	24/124 (19.4%)	5/86 (5.8%)
Recent infection	57/186 (30.6%)	61/298 (20.5%)	11/124 (8.9%)	6/86 (7.0%)
Hospitalized	34/186 (18.3%)	Not applicable	21/124 (16.9%)	Not applicable
Other acute infections				
Chikungunya virus	0/152 (0%)	Not applicable	53/123 (43.1%)	3/86 (3.5%)
Zika virus	Not applicable	Not applicable	0/123 (0%)	0/86 (0%)

DENV = dengue virus; IgM = immunoglobulin M; NS1 RT = nonstructural protein 1 rapid test; SD = standard deviation. Characteristics of index cases and associates in 2014 and 2015: mean age (SD) and gender, febrile status, hospitalization status, and arbovirus infection status (DENV acute infection: NS1 RT, NS1 ELISA, or reverse transcription polymerase chain reaction (RT-PCR) positive; DENV recent infection: IgM positive and NS1 RT/NS1 ELISA/RT-PCR negative; chikungunya virus and Zika virus confirmed by RT-PCR).

**Figure 3.**
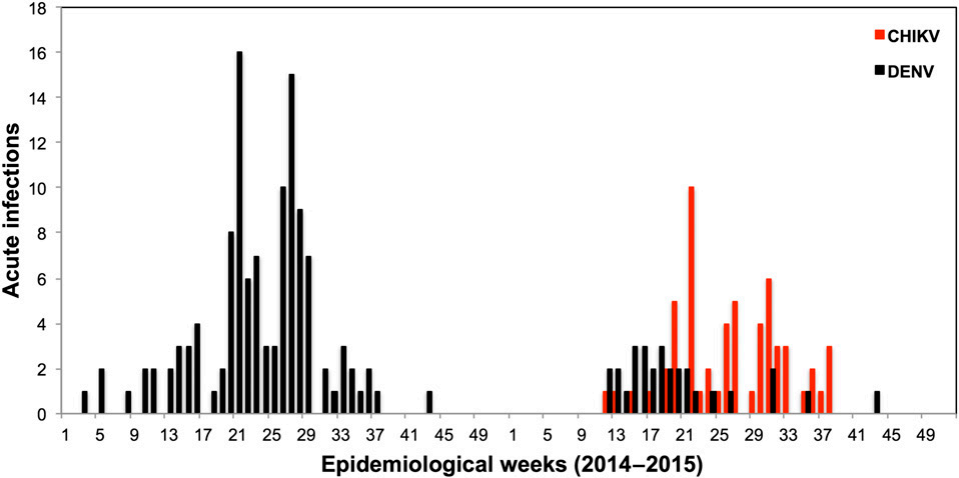
Weekly laboratory-confirmed acute dengue virus (DENV) and chikungunya virus (CHIKV) infections in 2014 and 2015 detected by passive and active surveillance. Note: no surveillance was conducted in week 30 of 2014. This figure appears in color at www.ajtmh.org.

Dengue virus transmission was highly seasonal in 2014 and 2015, with a peak in May (Figure 3). Chikungunya virus was first identified in our study on epidemiological week 12 in 2015 and transmission followed a similar seasonal curve as DENV (Figure 3). No ZIKV infections were detected (Table 1).

Table 1 shows the diagnostic results from 2014 and 2015. There were some individuals who did not have enough information to categorize as DENV positive or negative, for example, an individual who was negative by NS1 RT and RT-PCR but did not have any ELISA or serology test results. To account for these discrepancies, prevalence estimates include people for whom test results were available, as indicated by the denominators in the diagnostic results section of the table.

### Passive surveillance of index cases.

In 2014, the majority of all index cases (132/186, 70.9%) were positive for an acute or recent (AR) DENV infection (Table 1). All four DENV serotypes were detected and DENV2 was the predominant serotype (43/51, 84.3% of serotyped index cases) (Supplemental Table 4). One individual was positive for DENV1 and DENV2. Secondary DENV infections were most prevalent (73/99, 73.7% of index cases with serology and AR DENV infections) (Supplemental Table 5). Index cases with acute DENV infections were on average 20.7 years of age (standard deviations [SD] = 15.7) and 62.7% were male (Supplemental Table 6). The majority reported a fever within the last 7 days (97.3%), 21.3% had fever (> 38°C) on entering the study, and 16.0% were hospitalized.

In 2015, more index cases were positive for acute CHIKV infections (52/123, 43.1%) than for AR DENV infections (35/124, 28.3%). One index case was positive for both acute DENV and CHIKV infections and five index cases were positive for recent DENV and acute CHIKV infections, resulting in 11.5% (6/52) of CHIKV infections with AR DENV infections. Dengue virus 1 was the predominant serotype (14/23, 60.9% of serotyped index cases) (Supplemental Table 4). Significantly, more primary DENV infections were reported in 2015 than in 2014 (21/31, 67.7% of index cases with serology and AR DENV infections, *P* < 0.001, Supplemental Table 5). Index cases with acute DENV infections were on average 19.3 years of age (SD = 12.8) and 54.1% were female (Supplemental Table 6). All index cases with acute DENV infections reported a fever within the last 7 days, 41.7% had fever on entering the study, and 33.3% were hospitalized. There were no significant differences in the demographics, febrile symptoms, or hospitalization rates for index cases with acute DENV infections between 2014 and 2015 (Supplemental Table 6, *P* > 0.05).

**Figure 4.**
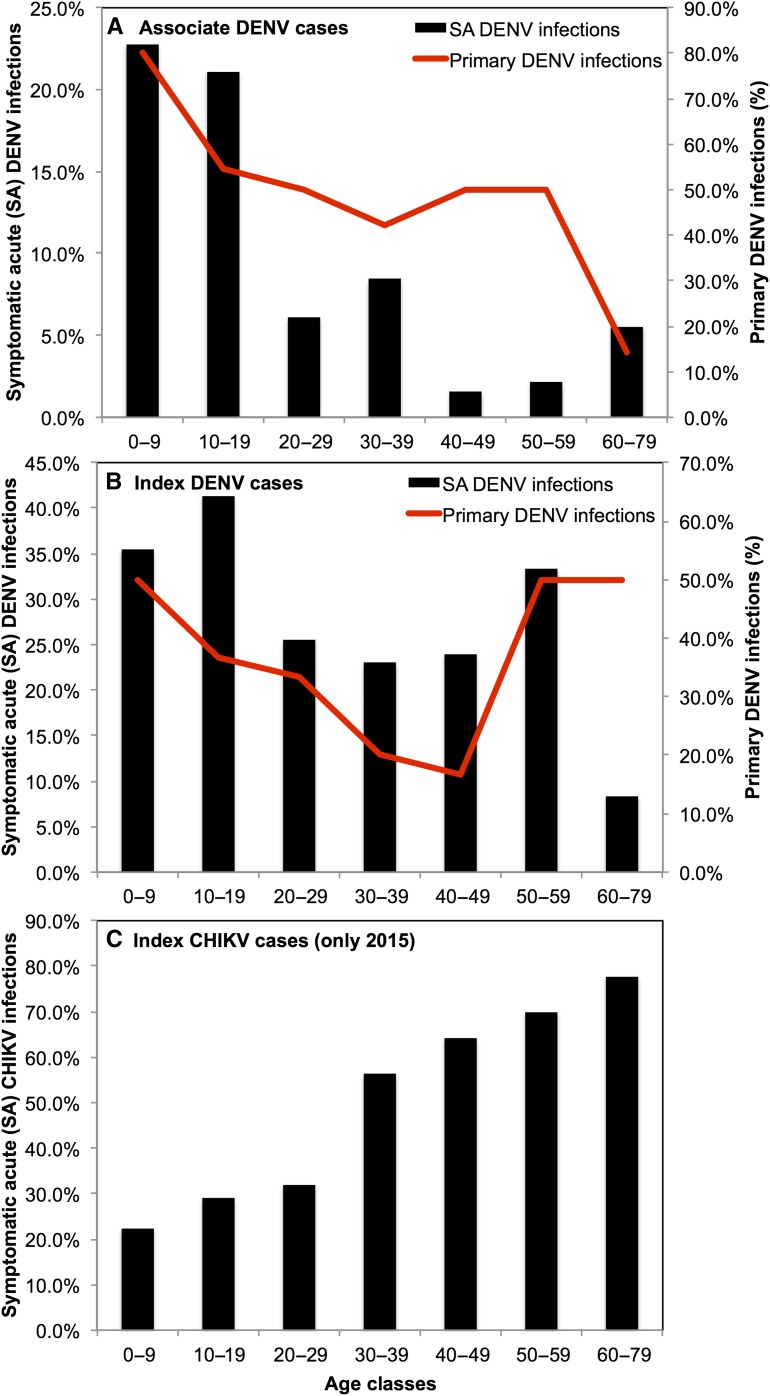
The prevalence of symptomatic acute (SA) infections and serology by age class. The prevalence of SA dengue virus (DENV) infections and the proportion of primary DENV infection in 2014 and 2015 for (**A**) associates and (**B**) index cases and (**C**) the prevalence of SA chikungunya virus (CHIKV) infections in index cases in 2015. See Supplemental Tables 7 and 8 for raw data and calculation details. This figure appears in color at www.ajtmh.org.

We estimated the prevalence of symptomatic acute (SA) infections for DENV and CHIKV by age class as a proportion of the total number of individuals recruited per age class (Figure 4, see Supplemental Tables 7 and 8 for prevalence calculations). Index children aged 10–19 years had the highest prevalence of SA DENV infections (40/97, 41.2%). Symptomatic acute DENV prevalence generally declined with increasing age, with the exception of individuals aged 50–59 years (7/21, 33.3%). Interestingly, the proportion of primary DENV infections decreased from 0 to 49 years and increased from 50 to 79 years (as determined by index cases with serology and AR DENV infections) although the sample size was small. By contrast, the prevalence of SA CHIKV infections, as a proportion of all individuals recruited into the study, was greatest in index cases aged 60–79 years (7/9, 77.8%) and prevalence increased with increasing age.

We compared the demographics and symptoms of index cases with acute DENV versus CHIKV infections (Table 2). Index cases with acute DENV infections were significantly younger (mean = 20.2 years, SD = 15.0) and more likely to report anorexia and nausea, vomiting, and abdominal pain (*P* < 0.05). Index cases with CHIKV were more likely to be female, were older (mean = 35.8 years, SD = 19.4), and were more likely to report muscle or joint pain (*P* < 0.05). A greater proportion of individuals with CHIKV reported rash (CHIKV: 34.6%, DENV: 16.5%; *P* = 0.05) and a lower proportion had fever (> 38°C) on entering the study (CHIKV: 11.8%, DENV: 26.5%; *P* = 0.06); however, these differences were not statistically significant.

**Table 2:** Demographics and symptoms associated with acute DENV infections vs. acute CHIKV infections in index cases

Characteristics	Acute DENV	Acute CHIKV	*P* value
	*N* = 98	*N* = 52	
Age in years, mean (SD)	20.2 (15.0)	35.8 (19.4)	**<0.0001**
Gender, % female	41/98 (41.8%)	35/52 (67.3%)	**0.005**
Temperature > 38°C	26/98 (26.5%)	6/51 (11.8%)	0.06
Hospitalized	20/98 (20.4%)	5/52 (9.6%)	0.14
Symptoms in prior 7 days			
Fever	97/98 (99.0%)	50/52 (96.2%)	0.57
Headache	80/97 (82.5%)	37/51 (72.5%)	0.23
Anorexia and nausea	64/98 (65.3%)	19/52 (36.5%)	**0.001**
Muscle/joint pain	75/97 (77.3%)	50/52 (96.2)	**0.006**
Rash	16/97 (16.5%)	18/52 (34.6%)	0.05
Bleeding	8/98 (8.2%)	2/52 (3.8%)	0.51
Vomiting	46/98 (46.9%)	12/52 (23.1%)	**0.007**
Drowsiness/lethargy	82/98 (93.9%)	46/52 (88.5%)	0.58
Abdominal pain	62/97 (63.9%)	19/52 (36.5%)	**0.002**
Diarrhea	27/98 (27.6%)	16/52 (30.8%)	0.82
Retro-orbital pain	67/98 (68.4%)	35/51 (68.6%)	1

CHIKV = chikungunya virus; DENV = dengue virus; SD = standard deviation. Index cases with acute DENV infections were significantly younger and more likely to report anorexia and nausea, vomiting, and abdominal pain (*P* < 0.05). Index cases with CHIKV were more likely to be female, were older, and were more likely to report muscle/joint pain (*P* < 0.05). One individual with a DENV and CHIKV coinfection was excluded. Bolded text denotes statistical significance (*P* <0.05).

We also compared the demographics and symptoms of primary versus secondary DENV infections (Supplemental Table 9) and DENV1 versus DENV2 infections in index cases (Supplemental Table 10). Individuals with secondary DENV infections were significantly older (secondary: mean = 23.2 years, SD = 13.8; primary: mean = 18.0 years, SD = 13.1) (*P* < 0.05). Overall, we identified more severe illness in secondary DENV infections; individuals with secondary infections were more likely to report vomiting and hospitalized individuals were more likely to have secondary DENV infections (*P* < 0.05). However, individuals with primary DENV infections were more likely to report fever (*P* < 0.05). We did not find significant differences in symptoms between DENV1 and DENV2 (*P* > 0.05), the predominant serotypes detected in this study, although index cases with DENV2 infections were significantly older (DENV1: mean = 14.7 years, SD = 10.5; DENV2: mean = 25.2 years, SD = 16.2) (*P* < 0.05).

### Active surveillance of associates.

In each cluster of homes, approximately nine associates were recruited into this study per initiate index case (Figure 2). The distance between the households of associates and the respective initiate index households ranged from 2.2 to 164 m, with an average of 39 m (SD = 29 m). Most associate households (95.4%) were within 100 m of the initiate index household.

In 2014, approximately one-third of all associates (106/298, 35.6%) had evidence of AR DENV infections (Table 1). As with index cases, DENV2 was the dominant serotype (Supplemental Table 4). A similar proportion of primary (46.9%) and secondary infections (53.0%) were detected (as determined by associates with serology and AR DENV infections) (Supplemental Table 5). In 2015, as with index cases, the prevalence of DENV infections decreased as a proportion of all associates recruited (11/86, 12.9%) and primary DENV infections were more common (4/6, 66.7% of associates with serology and AR DENV infections, Supplemental Table 5). Only one associate was serotyped as DENV2 (Supplemental Table 4). The serology of associates in 2014 versus 2015 was not significantly different due, in part, to the small sample size (*P* > 0.05). In 2015, we detected acute CHIKV infections in three associates (3/86, 3.5%), including one associate with both acute CHIKV and recent DENV infections.

Approximately two-thirds of associates with acute DENV infections (34/50, 68%) reported one or more dengue-like symptoms within the last 7 days, resulting in a ratio of symptomatic:inapparent infections (S:I) of 1:0.47 (2.13) (Supplemental Table 1). The most commonly reported symptoms were headache (32%), drowsiness/lethargy (24%), fever (22%), muscle/joint pain (22%), and retro-orbital pain (22%). Only two associates with SA DENV infections had sought medical care within the last 7 days (2/34, 5.9%) and no associates were hospitalized because of a DENV infection (Supplemental Table 6). There were no significant differences in the demographics or febrile symptoms of associates with acute DENV infections in 2014 versus 2015 (*P* > 0.05, Supplemental Table 6).

In associates, we determined the prevalence of SA DENV infections by age class as a proportion of the total number of associates recruited per age class (Figure 4, Supplemental Tables 7 and 8). Children aged 0–9 years had the highest prevalence of SA DENV infections (5/22, 22.7%) and prevalence declined with increasing age. The proportion of primary DENV infections similarly decreased with increasing age. We calculated the prevalence of symptomatic infections in associates with positive primary and secondary DENV infections and found that individuals with secondary infections had a higher prevalence of symptomatic disease; however, the differences were not statistically significant (symptomatic primary: 24/42, 57.1%; symptomatic secondary 35/45, 77.8%; *P* = 0.07). No associates had SA CHIKV infections.

At the cluster level, prevalence rates varied by the DENV serotype of the initiate index case. In 10 of 44 clusters, the initiate index case had a DENV1 infection. In these clusters, 20% of all associates had AR DENV infections (12/60; 95% confidence interval [CI]: 11.8–31.8%), with a range of 0–57.1%. The initiate index case had a DENV2 infection in 17 of 44 clusters. Among these clusters, a significantly greater proportion of all associates (36.6%; 59/161; 95% CI: 29.6–44.3%) (*P* = 0.02) had an AR DENV infections, with a range of 12.5% to 87.5%.

We calculated the average number of AR DENV infections and symptomatic acute and recent (SAR) infections per cluster (see raw data in Supplemental Table 11). By definition, each cluster included an initiate index case, which was a SAR infection. In 2014, there were 32 clusters, with an average of 10.3 (SD = 2.7) individuals enrolled per cluster. We detected an average of 4.3 (SD = 2.3) AR infections, of which 3.3 (SD = 1.7) were SAR infections per cluster. In 2015, there were 12 clusters, with an average of 8.2 (SD = 2.2) individuals enrolled per cluster. We detected an average of 1.9 (SD = 0.7) AR infections, of which 1.4 (SD = 0.7) were SAR infections. All measures were significantly greater in 2014 than in 2015 (*P* < 0.05). Over both years, we detected an average of 3.7 (SD = 2.3) AR infections and 2.8 (SD = 1.7) SAR infections per cluster.

### Phylogenetic analysis of DENV.

**Figure 5.**
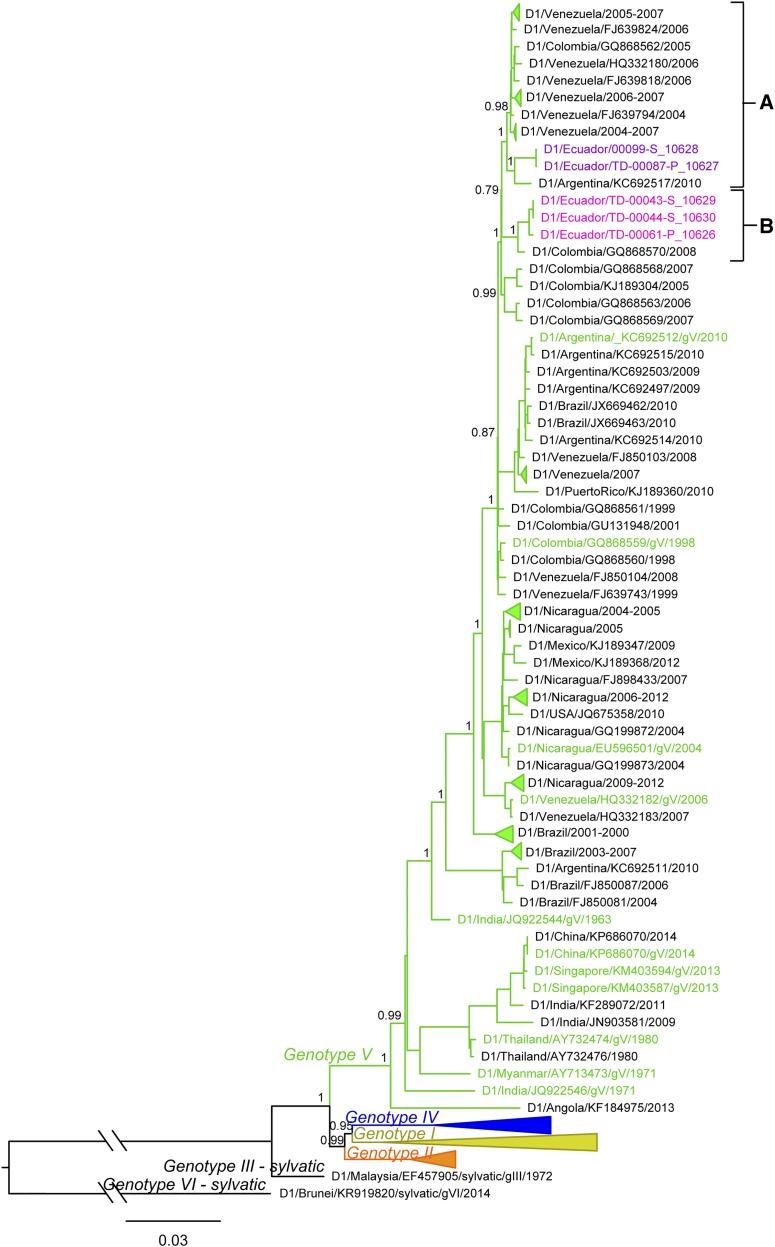
Maximum likelihood phylogenetic tree of dengue virus 1 genotypes from Ecuador in 2014. Samples from Ecuador are colored in magenta (dark and light). The two clades containing the genomes from Ecuador are marked in the tree (A and B). Approximate likelihood ratio test (aLRT) confidence values are shown next to the respective node. The tree is rooted on the sylvatic genotype *VI* sample. Some clades were collapsed in the tree to increase clarity. All collapsed clades were supported with high (> 0.75) aLRT values and contained only genomes from a single country, indicated in the name of the clade. Colored taxa represent known genotype references. This figure appears in color at www.ajtmh.org.

The best-fit models for the evolution of DENV1, DENV2, and DENV4, as determined by AIC versus BIC, agreed in all instances. Maximum likelihood phylogenetic tree demonstrated a clear distinction of DENV1 genotypes *I*, *II*, *IV*, and *V*, and the sylvatic genotypes *III* and *VI* (Figure 5). The five genomes from Ecuador, all sampled in 2014, belonged to genotype *V* of DENV1 and were found in the sub-lineage containing mainly Central and South American genomes (i.e., Colombia, Venezuela, Argentina, Brazil, and Puerto Rico). More importantly, sequences from Ecuador fell into two distinct clades within this sub-lineage; two Ecuadorian genomes were more closely related to genomes sampled in Argentina and Venezuela (clade A) and three Ecuadorian genomes were more closely related to a genome from Colombia (clade B).

**Figure 6.**
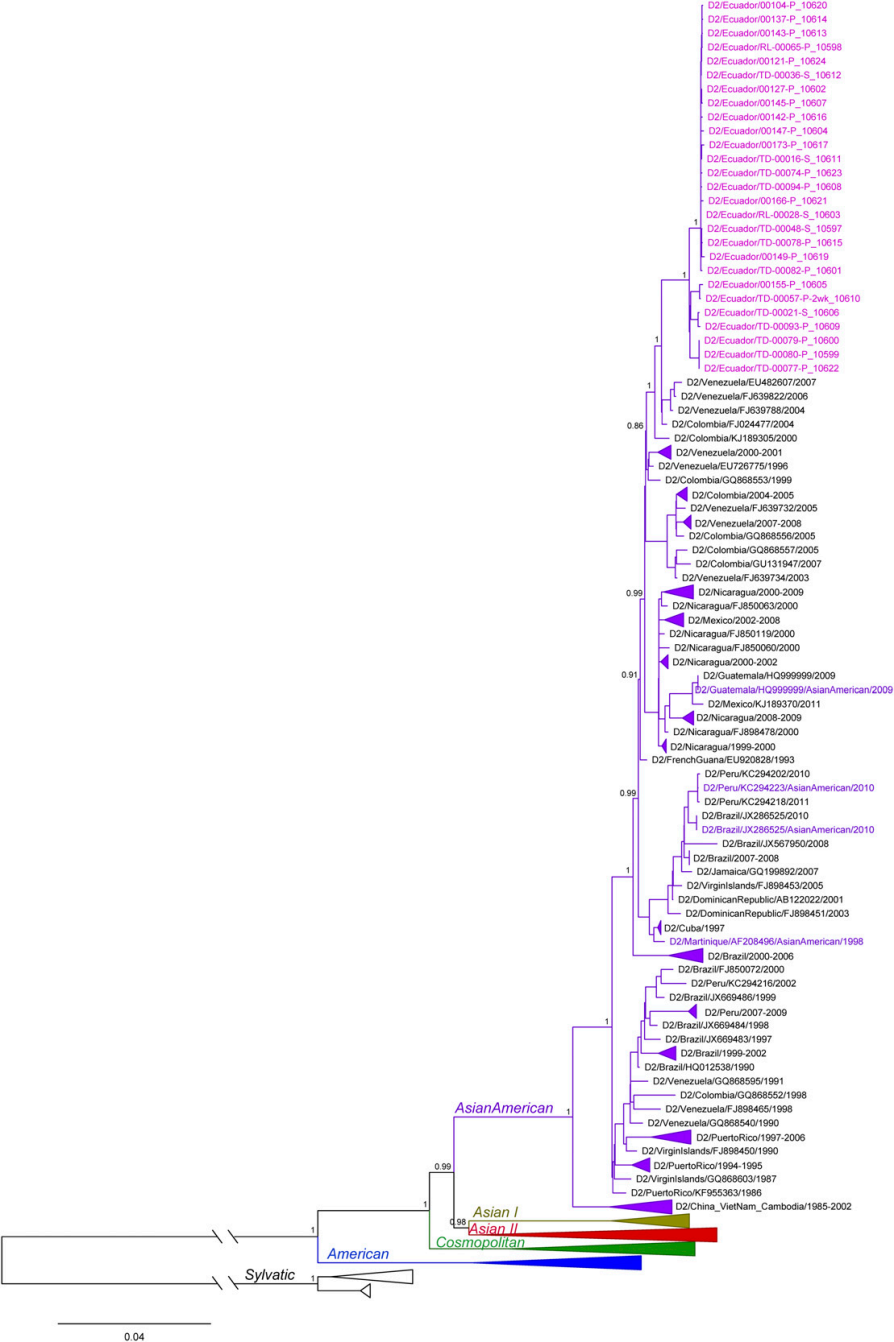
Maximum likelihood phylogenetic tree of dengue virus 2 genotypes from Ecuador in 2014. Samples from Ecuador are colored in magenta in a monophyletic clade A. Approximate likelihood ratio test (aLRT) confidence values are shown next to the respective node. The tree is rooted on the sylvatic genotype out-group. Some clades were collapsed in the tree to increase clarity. All collapsed clades were supported with high (> 0.75) aLRT values and contained only genomes from a single country, indicated in the name of the clade. Colored taxa represent known genotype references. This figure appears in color at www.ajtmh.org.

The ML phylogenetic tree of DENV2 showed a clear distinction of DENV2 genotypes, including sylvatic, American, Cosmopolitan, Asian I, Asian II, and Asian/American (Figure 6). The samples from Ecuador were found within the Asian/American genotype, making up a monophyletic cluster (clade A) separated from the rest of the South American taxa with high support (aLRT = 1). Genomes clustering closest to the clade A from Ecuador were sampled in Colombia and Venezuela. Sequences from other neighboring countries, such as Peru and Brazil, were found further down in the Asian/American lineage and were separated from the clade A, and from sequences from Colombia and Venezuela, with high support (aLRT = 0.99).

**Figure 7.**
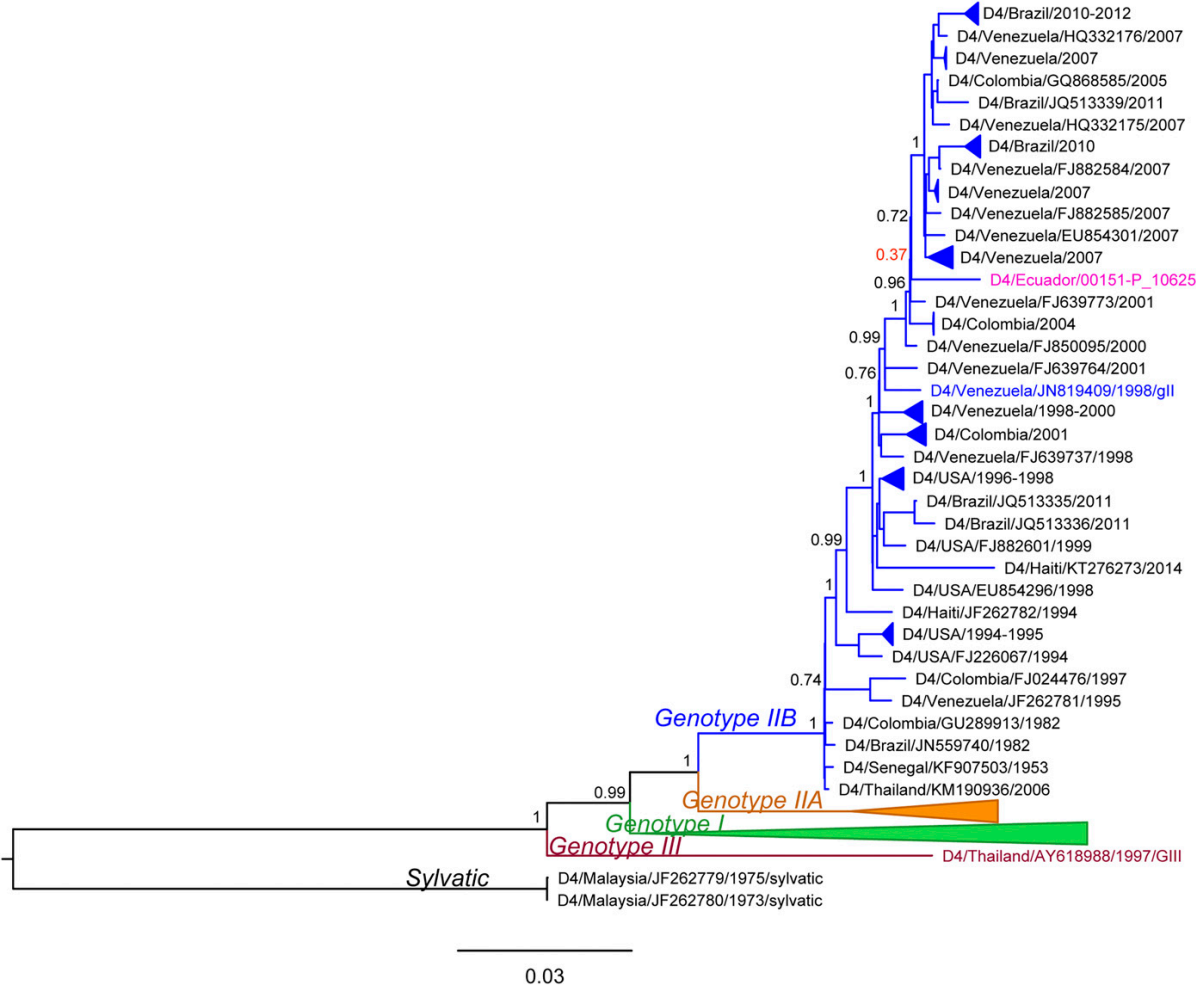
Maximum likelihood phylogenetic tree of dengue virus 4 genotypes from Ecuador in 2014. Sample from Ecuador is colored in magenta. Approximate likelihood ratio test (aLRT) confidence values are shown next to the respective node. Low aLRT values are highlighted in red. The tree is rooted on the sylvatic genotype out-group. Some clades were collapsed in the tree to increase clarity. All collapsed clades were supported with high (> 0.75) aLRT values and contained only genomes from a single country, indicated in the name of the clade. Colored taxa represent known genotype references. This figure appears in color at www.ajtmh.org.

The ML phylogenetic tree of DENV4 demonstrated a clear distinction of genotypes *I*, *IIA*, *IIB*, *III*, and sylvatic (Figure 7). However, two taxa from India/1961–1962 clustered with genotype *I* with low support (aLRT = 0.04), indicating that their position in the tree was uncertain and they might belong to a different genotype. The single Ecuador sequence was located within the genotype *IIB* lineage (magenta in the tree). It was surrounded by sequences collected from Venezuela, Colombia, and Brazil, indicating their common ancestry. However, the aLRT support for the Ecuador node was low (0.4), suggesting that its correct placement was uncertain.

## DISCUSSION

In this study, we characterized the epidemiology and clinical characteristics of DENV and CHIKV infections, and the phylogenetics of DENV through an enhanced surveillance study design in an endemic region. We found that the burden of SA DENV in associates was greatest in children aged less than 10 years. In 2014, for every SA DENV infection detected by passive surveillance (initiate index cases), we detected an additional three AR DENV infections in associates by active surveillance. Two-thirds of associates with acute DENV infections presented with dengue-like symptoms. The prevalence of DENV decreased from 2014 to 2015 with the emergence of CHIKV. Genetic analyses indicate that there is movement of the DENV between Ecuador and neighboring countries, highlighting the importance of sentinel surveillance sites, such as Machala, in border regions. The rapid surveillance methods developed in this study could be applied to estimate the burden of other underreported febrile diseases, allowing the public health sector to more effectively and equitably conduct disease control interventions.

### Burden of DENV infection.

Over the 2 years of the study, one-third of associates had AR DENV infections, a higher prevalence than findings from similar studies in Asia. In Vietnam, studies found 18% DENV prevalence in 100-m clusters around initiate index cases, using RT-PCR, NS1 ELISA, or serology.^21^ In Thailand, cluster DENV prevalence ranged from 10.1% to 14.3% using RT-PCR or serology.^22,23^ One of the possible explanations for the higher cluster prevalence in this study is the use of the NS1 RT. Prior studies that evaluated the Panbio Dengue Early Rapid test (used in this study) found that using antigen (NS1) and antibody (IgM and IgG) tests together increased the sensitivity of DENV diagnostics (93% sensitivity) and expanded the window of detection of infection.^52^ We found that the prevalence of DENV infections in clusters varied by DENV serotype (DENV1: 20.0%, DENV2: 36.6%). The higher cluster prevalence for DENV2 is consistent with prior studies that found greater infection rates for DENV2 compared with DENV1.^53^ The cause of the difference in infection rates between the two serotypes is not understood. Potential factors that could be involved include the local epidemiology, serotype subtype, weather, and previous exposure history of the population.^54–56^

Using this active cluster surveillance protocol, we were able to effectively detect additional DENV infections in the community, particularly in 2014, when there was a higher burden of disease. For every initiate index case captured by passive surveillance, we captured approximately three associates with AR DENV infections, of which two associates had SAR DENV infections. Interestingly, we found that the number of DENV infections per cluster was higher in 2014 than 2015, suggesting a higher force of DENV infection in 2014, when all four DENV serotypes were circulating, before the emergence of CHIKV. We temper this with caution, however, as our cluster sample size was smaller in 2015 (*N* = 12) than in 2014 (*N* = 32).

In Latin America, enhanced surveillance studies that have reported DENV infection rates relative to passive surveillance infection rates include pediatric and adult cohorts, door-to-door community-based surveillance studies, use of sentinel clinics, and enhanced laboratory diagnostic studies. To our knowledge, most cluster-based DENV surveillance studies with a similar design (e.g., spatially restricted around the index home) have been conducted in Asian countries. Estimates of the burden of disease from active surveillance studies in Latin America vary widely depending on the study design, the effectiveness of passive surveillance, and the traits of the local population (e.g., past exposure to DENV serotypes). In a pediatric cohort in Nicaragua, investigators detected 21.3 times more DENV infections than were reported to the national surveillance system.^57^ A study in Peru compared passive surveillance of DENV to a cohort study and sentinel clinic surveillance and found five times more DENV infections in the cohort and 19 times more DENV infections through sentinel clinic surveillance.^25^ They found that both sentinel and cohort surveillance methods detected an increase in DENV infections more rapidly than passive surveillance methods. In Puerto Rico, laboratory-enhanced surveillance resulted in three times more DENV infections registered than passive surveillance methods.^27^

One of the limitations of this study was that we surveyed the nearest neighbors of the initiate index case, which are not necessarily representative of the total population residing within 200 m. We did not collect information on those who were not willing to participate in the study. Also, people may have been more willing to participate in the study if they or someone in their household was ill. This could potentially result in a higher estimate of the number of additional DENV infections in clusters compared with the general population. Future studies could survey a greater number of households located randomly within the 200-m radius for a more accurate measure of disease prevalence and could assess DENV-negative clusters as controls. In addition, this study was conducted in clinical sites operated by the MoH that were willing and able to support the study. Testing for CHIKV and ZIKV was done by RT-PCR and did not include serological testing.

### Burden of CHIKV and other febrile illness.

In 2015, we found that 43.1% of clinically diagnosed (suspected) DENV infections were actually positive for CHIKV, higher than the proportion of laboratory-confirmed DENV infections. We identified six index cases and one associate with evidence of both acute CHIKV and AR DENV infections in 2015 (11.5% of CHIKV infections). There were also 96 individuals with undiagnosed febrile illness (non-DENV, non-CHIKV, and non-ZIKV). The burden of CHIKV is likely higher than reported here because we only tested for acute infections. This highlights the difficulties of differential diagnosis in areas where DENV, CHIKV, ZIKV, and other febrile illnesses are co-circulating. These data also suggest that the large increase in DENV cases in 2015 in Ecuador (44,104 cases in 2015 versus 14,312 cases on average from 2010 to 2014)^11^ could be the result of CHIKV and other circulating febrile pathogens.

We did not detect ZIKV during the study period, consistent with MoH reports, which indicated that ZIKV circulated for the first time in Machala in February 2016. Although surveillance efforts were not focused specifically on clinical ZIKV infections, we suspect that the study would have detected some ZIKV infections if they were present in Machala due to the overlapping clinical presentations of DENV and ZIKV infections. However, recent studies indicate that urine and whole blood may be better suited to detect ZIKV, limiting our ability to detect ZIKV in serum samples by RT-PCR.^58,59^

### Clinical characteristics of DENV and CHIKV infections.

In general, the symptoms that were observed with acute DENV infections in this study are consistent with other reports.^60–66^ As in other studies, we found that secondary DENV infections were more severe; nine of 10 hospitalized individuals with DENV infections had secondary infections (Supplemental Table 9).^24,65,67^ From 2014 to 2015, we observed a shift from DENV2 to DENV1 and a shift from secondary to primary DENV infections. As expected, associates with acute DENV infections in 2015 were younger (mean = 19.6 years of age) than in 2014 (mean = 25.2 years of age), although the differences were not significantly different (Supplemental Table 6). The clinical characteristics associated with DENV infections can vary over time and space because of both differences in the dominant serotypes in circulation^68,69^ and the ratio of primary to secondary infections.^24,65,67^

People infected with CHIKV versus DENV were older on average, consistent with the disease being newly introduced into the population. Ministry of Health reports indicated that the highest burden of CHIKV in Machala was among adults aged 20–49. We found that muscle and joint pain and rash were more commonly reported by people with CHIKV infections than those with DENV, which supports findings from prior studies.^62,66^

The ratio of S:I DENV infections in associates was 1:0.47 (2.13), which is within the upper range of prior estimates from DENV endemic regions. By defining symptomatic as any dengue-like symptom, rather than only fever, we captured a broad spectrum of DENV illness. Prior studies suggest that the S:I ratio for DENV infections can vary widely, possibly depending on the immune response to prior exposure to DENV serotypes, the serotypes (and subtypes) in circulation, and genetic factors.^23,24,31,33,69,70^ A 1-year contact cluster study from Peru reported an S:I ratio of 1:4.56 (0.22).^31^ A 4-year pediatric cohort study from Nicaragua reported S:I ratios ranging from 1:18.4 (0.05) to 1:3.0 (0.33).^69^ Symptomatic:inapparent ratios from a 5-year school cohort study in Thailand ranged from greater than 4 to 0, depending on the year and school.^33,70^ A 2-year school cohort and cluster study from Thailand reported an overall S:I ratio of 1:1 (1.0)^23^ and a 1-year cluster surveillance study from Thailand reported 1:0.2 (5.0) for primary infections and 1:0.4 (2.5) for secondary infections.^23^ Differences may also be due to the profile of the study population (e.g., adult versus pediatric) and how investigators defined symptomatic.

Despite the high proportion of associates with SA DENV infections, few (5.9%) had sought medical care. In prior studies in Machala, community members and health-care professionals indicated that there was low health-care–seeking behavior in certain populations, such as working men in the urban periphery, and self-medication was the common practice.^18,71^ Another explanation is that our definition of symptomatic DENV infections included mildly symptomatic infections that did not require medical attention. These findings highlight the importance of active surveillance protocols that capture inapparent infections and infections in demographic groups who are less likely to seek health care or who have limited access to health care.

### Phylogenetic analysis.

Phylogenetic analyses of DENV1 showed Ecuadorian samples falling into two distinct clusters, sharing a common ancestor with viruses from Colombia in one cluster and a common ancestor with viruses from Venezuela in the other cluster. These well-separated clusters indicate at least two distinct introductions of DENV1 into Ecuador. Given the early sampling of Venezuelan and Colombian genomes (between 2004 and 2008) and given that recent DENV1 full-genome samples from Peru are not available, we cannot exclude with certainty the role that Peru may have played in the DENV1 introductions into Ecuador. However, the results suggest a close genetic relationship of viruses circulating in Venezuela and Colombia and support the notion of commonly occurring DENV1 flow between the countries. Similar to DENV1, DENV2 genomes from Ecuador were most closely related to genomes from Venezuela and Colombia. However, unlike DENV1, DENV2 genomes from Ecuador made up a single monophyletic clade separated from the rest of the South American taxa with high support. This indicates a single introduction and subsequent spread of this virus in Ecuador without further DENV2 introductions and mixing from other regions. Even though older sequences from Peru clustered further away from genomes sampled in Ecuador, Venezuela, and Colombia, suggesting they did not play a role in the current DENV2 epidemic in Ecuador, the lack of recent full genomes from Peru prevent us from determining the involvement of Peru in the observed DENV2 spread in Ecuador. The unavailability of recent full genomes from countries surrounding Ecuador was most evident in DENV4, where the exact placement of the only Ecuadorian genome in the tree could not be determined because of low node support. Nevertheless, the results suggested a close relationship between DENV4 in Ecuador, Venezuela, Colombia, and Brazil. It is important to note that samples from Peru were missing here as well and that there is a possibility this country was also involved in the circulation of DENV4 in this region. Thus, our results suggest frequent flow of DENV between Ecuador and surrounding countries, including introduction and reintroduction of different serotypes and different lineages of the same serotype. In addition, our results show the importance of continuous surveillance, including genetic sequencing efforts. If available, virus full genomes from these countries would allow for more accurate analysis of the patterns of DENV movement and spread in this region.

### Public health implications.

This study provides one of the most thorough descriptions of DENV and CHIKV infections in this region and contributes to a long-term collaboration with the MoH and other governmental and academic partners to strengthen infectious disease surveillance in southern coastal Ecuador, a strategic area to monitor endemic and emerging pathogens. The collaboration has been successful because of a shared vision for integrated active surveillance that includes the virus–vector–host, climate, and other social–ecological drivers;^20,32^ ongoing training of physicians, researchers, and students; and improvement of local diagnostic and research infrastructure.

Enhanced surveillance studies, such as this, provide high-resolution spatiotemporal data on the distribution of symptomatic and inapparent infections across the population. This is especially important in places and in subgroups with low health-care–seeking behavior, which result in underreporting and continued disease transmission.^18,71^ Enhanced surveillance systems have been shown to detect an increase in infections earlier than passive surveillance systems,^25^ providing a warning of an escalating outbreak. These data are presently being used to parameterize and calibrate local epidemic forecast models.^72,73^ These data also allow the public health sector to more accurately estimate the social and economic cost of the disease, allowing for informed decision-making regarding the allocation of scarce resources for current and future interventions, such as vector control, community mobilization, and vaccines.^74,75^ The age-stratified prevalence data generated through this study design provides important information for the design of future vaccine trials and vaccination campaigns.

Genetic and phylogenetic analyses provided insights regarding about virus movement and introductions into Ecuador. Determining sources of viral origin and most common pathways of spread provides important information about the dynamics of the epidemic that can aid in development of coordinated regional public health surveillance and control efforts, especially across Andean countries.^76^ In addition, frequent movement of dengue between Ecuador and neighboring countries highlighted the critical role of sentinel surveillance sites, such as Machala, in border regions. Finally, recent reports have documented for the first time the presence of *Aedes albopictus*, the Asian tiger mosquito, in the nearby city of Guayaquil, Ecuador.^77^ Efforts are needed to monitor the emergence and spread of the mosquito vector and its role in arbovirus transmission.

## Supplementary Material

Supplemental Tables

## References

[1] WHO, 2009 *Dengue: Guidelines for Diagnosis, Treatment, Prevention and Control.* Geneva, Switzerland: World Health Organization. Available at: http://www.who.int/rpc/guidelines/9789241547871/en/. Accessed December 6, 2017. 23762963

[2] Dick OB Martín JLS Montoya RH del Diego J Zambrano B Dayan GH, 2012 The history of dengue outbreaks in the Americas. Am J Trop Med Hyg 87: 584–593. 23042846 10.4269/ajtmh.2012.11-0770 PMC3516305

[3] San Martín JL Brathwaite O Zambrano B Solórzano JO Bouckenooghe A Dayan GH Guzmán MG, 2010 The epidemiology of dengue in the Americas over the last three decades: a worrisome reality. Am J Trop Med Hyg 82: 128–135. 20065008 10.4269/ajtmh.2010.09-0346 PMC2803522

[4] Stanaway JD 2016 The global burden of dengue: an analysis from the Global Burden of Disease Study 2013. Lancet Infect Dis 16: 712–723. 26874619 10.1016/S1473-3099(16)00026-8 PMC5012511

[5] Bhatt S Gething PW Brady OJ Messina JP Farlow AW Moyes CL Drake JM Brownstein JS Hoen AG Sankoh O, 2013 The global distribution and burden of dengue. Nature 496: 504–507. 23563266 10.1038/nature12060 PMC3651993

[6] WHO, 2017 *Dengue and Severe Dengue.* Geneva, Switzerland: World Health Organization. Available at: http://www.who.int/mediacentre/factsheets/fs117/en/. Accessed December 6, 2017.

[7] PAHO/WHO, 2017 *Number of Reported Cases of Chikungunya Fever in the Americas, by Country or Territory.* Geneva, Switzerland: World Health Organization. Available at: http://www.paho.org/hq/index.php?option=com_topics&view=readall&cid=5927&Itemid=40931&lang=en. Accessed February 16, 2018.

[8] Zanluca C Melo VC Mosimann AL Santos GI Santos CN Luz K, 2015 First report of autochthonous transmission of Zika virus in Brazil. Mem Inst Oswaldo Cruz 110: 569–572. 26061233 10.1590/0074-02760150192 PMC4501423

[9] Campos GS Bandeira AC Sardi SI, 2015 Zika virus outbreak, Bahia, Brazil. Emerg Infect Dis 21: 1885–1886. 26401719 10.3201/eid2110.150847 PMC4593454

[10] PAHO/WHO, 2017 *Zika Cases and Congenital Syndrome Associated with Zika Virus Reported by Countries and Territories in the Americas.* Cumulative Cases. Washington, DC: Pan American Health Organization/World Health Organization. Available at: http://www.paho.org/hq/index.php?option=com_content&view=article&id=12390&Itemid=42090&lang=en. Accessed February 16, 2018.

[11] PAHO/WHO, 2017 *Annual Cases Reported of Dengue.* Data, Maps and Statistics. Washington, DC: Pan American Health Organization/World Health Organization. Available at: http://www.paho.org/hq/index.php?option=com_topics&view=rdmore&cid=6290&Itemid=40734. Accessed July 18, 2017.

[12] Camargo S, 1967 History of Aedes aegypti eradication in the Americas. Bull World Health Organ 36: 602. 5299460 PMC2476393

[13] Gonzalez V Jurado H, 2007 *Guayaquil:* Aedes aegypti*, 1740–2007*. Guayaquil, Ecuador: Servicio Nacional para La Eradicaccion de Malaria (SNEM) of the Ministry of Health of Ecuador.

[14] United States Centers for Disease Control (CDC), 1989 Dengue epidemic—Ecuador, 1988. MMWR Morb Mortal Wkly Rep 38: 419–421. 2498633

[15] Alava A Mosquera C Vargas W Real J, 2005 Dengue en el Ecuador 1989–2002. Rev Ecuat Hig Med Trop 42: 11–34.

[16] Stewart-Ibarra AM Lowe R, 2013 Climate and non-climate drivers of dengue epidemics in southern coastal Ecuador. Am J Trop Med Hyg 88: 971–981. 23478584 10.4269/ajtmh.12-0478 PMC3752767

[17] Stewart-Ibarra AM Ryan SJ Beltrán E Mejía R Silva M Muñoz Á, 2013 Dengue vector dynamics (Aedes aegypti) influenced by climate and social factors in Ecuador: implications for targeted control. PLoS One 8: e78263. 24324542 10.1371/journal.pone.0078263 PMC3855798

[18] Stewart-Ibarra AM Luzadis VA Borbor-Cordova MJ Silva M Ordonez T Beltran Ayala E Ryan SJ, 2014 A social-ecological analysis of community perceptions of dengue fever and Aedes aegypti in Machala, Ecuador. BMC Public Health 14: 1135. 25370883 10.1186/1471-2458-14-1135 PMC4240812

[19] Stewart Ibarra AM Muñoz AG Ryan SJ Borbor MJ Ayala EB Finkelstein JL Mejia R Ordonez T Coronel GCR Rivero K, 2014 Spatiotemporal clustering, climate periodicity, and social-ecological risk factors for dengue during an outbreak in Machala, Ecuador, in 2010. BMC Infect Dis 14: 610. 25420543 10.1186/s12879-014-0610-4 PMC4264610

[20] Kenneson A Beltrán-Ayala E Borbor-Cordova MJ Polhemus ME Ryan SJ Endy TP Stewart-Ibarra AM, 2017 Social-ecological factors and preventive actions decrease the risk of dengue infection at the household-level: results from a prospective dengue surveillance study in Machala, Ecuador. PLoS Negl Trop Dis 11: e0006150. 29253873 10.1371/journal.pntd.0006150 PMC5771672

[21] Anders KL 2015 Households as foci for dengue transmission in highly urban Vietnam. PLoS Negl Trop Dis 9: e0003528. 25680106 10.1371/journal.pntd.0003528 PMC4332484

[22] Yoon I-K 2012 Fine scale spatiotemporal clustering of dengue virus transmission in children and *Aedes aegypti* in rural Thai villages. PLoS Negl Trop Dis 6: e1730. 22816001 10.1371/journal.pntd.0001730 PMC3398976

[23] Mammen MP 2008 Spatial and temporal clustering of dengue virus transmission in Thai villages. PLoS Med 5: e205. 18986209 10.1371/journal.pmed.0050205 PMC2577695

[24] Thomas SJ 2015 Improving dengue virus capture rates in humans and vectors in Kamphaeng Phet province, Thailand, using an enhanced spatiotemporal surveillance strategy. Am J Trop Med Hyg 93: 24–32. 25986580 10.4269/ajtmh.14-0242 PMC4497898

[25] Olkowski S Stoddard ST Halsey ES Morrisson AC Barker CM Scott TW, 2016 Sentinel versus passive surveillance for measuring changes in dengue incidence: evidence from three concurrent surveillance systems in Iquitos, Peru. bioRxiv 040220, 10.1101/040220.

[26] Rocha C Morrison AC Forshey BM Blair PJ Olson JG Stancil JD Sihuincha M Scott TW Kochel TJ, 2009 Comparison of two active surveillance programs for the detection of clinical dengue cases in Iquitos, Peru. Am J Trop Med Hyg 80: 656–660. 19346395

[27] Ramos MM Argüello DF Luxemburger C Quiñones L Muñoz JL Beatty M Lang J Tomashek KM, 2008 Epidemiological and clinical observations on patients with dengue in Puerto Rico: results from the first year of enhanced surveillance—June 2005–May 2006. Am J Trop Med Hyg 79: 123–127. 18606775

[28] Restrepo BN Piedrahita LD Agudelo IY Parra-Henao G Osorio JE, 2012 Frequency and clinical features of dengue infection in a schoolchildren cohort from Medellin, Colombia. J Trop Med 2012 Available at: https://www.hindawi.com/journals/jtm/2012/120496/abs/. Accessed May 11, 2017. 10.1155/2012/120496 PMC3530854 23304167

[29] Espino C, 2010 *Active Surveillance and Incidence Rate of Dengue Infection in a Cohort of High Risk Population in Maracay, Venezuela* Tampa, FL: University of South Florida. Available at: http://scholarcommons.usf.edu/etd/1626/. Accessed May 11, 2017.

[30] Kuan G Gordon A Avilés W Ortega O Hammond SN Elizondo D Nuñez A Coloma J Balmaseda A Harris E, 2009 The Nicaraguan pediatric dengue cohort study: study design, methods, use of information technology, and extension to other infectious diseases. Am J Epidemiol 170: 120–129. 19435864 10.1093/aje/kwp092 PMC2700880

[31] Stoddard ST 2013 House-to-house human movement drives dengue virus transmission. Proc Natl Acad Sci USA 110: 994–999. 23277539 10.1073/pnas.1213349110 PMC3549073

[32] Borbor-Cordova M 2016 Case study 5.C vector-virus microclimate surveillance system for dengue control in Machala, Ecuador. *Climate Services for Health: Improving Public Health Decision-Making in a New Climate* Geneva, Switzerland: World Meteorological Association and World Health Organization. Available at: http://public.wmo.int/en/resources/library/climate-services-health-case-studies. Accessed September 3, 2016.

[33] Endy TP Chunsuttiwat S Nisalak A Libraty DH Green S Rothman AL Vaughn DW Ennis FA, 2002 Epidemiology of inapparent and symptomatic acute dengue virus infection: a prospective study of primary school children in Kamphaeng Phet, Thailand. Am J Epidemiol 156: 40–51. 12076887 10.1093/aje/kwf005

[34] Sommerfeld J Kroeger A, 2012 Eco-bio-social research on dengue in Asia: a multicountry study on ecosystem and community-based approaches for the control of dengue vectors in urban and peri-urban Asia. Pathog Glob Health 106: 428–435. 23318234 10.1179/2047773212Y.0000000055 PMC3541880

[35] Quintero J 2014 Ecological, biological and social dimensions of dengue vector breeding in five urban settings of Latin America: a multi-country study. BMC Infect Dis 14: 38. 24447796 10.1186/1471-2334-14-38 PMC3904013

[36] Ministerio de Salud Publica, 2010 *Casos de Dengue Reportados En El Epi Local Por Semanas Epidemiologicas*. Machala, Ecuador: Departamento de Epidemiologia, Direccion Provincial de Salud de El Oro, Ministerio de Salud Publica.

[37] Pan-ngum W Blacksell SD Lubell Y Pukrittayakamee S Bailey MS de Silva HJ Lalloo DG Day NPJ White LJ Limmathurotsakul D, 2013 Estimating the true accuracy of diagnostic tests for dengue infection using Bayesian latent class models. PLoS One 8: e50765. 23349667 10.1371/journal.pone.0050765 PMC3548900

[38] Pal S 2015 Multicountry prospective clinical evaluation of two enzyme-linked immunosorbent assays and two rapid diagnostic tests for diagnosing dengue fever. J Clin Microbiol 53: 1092–1102. 25588659 10.1128/JCM.03042-14 PMC4365197

[39] United States Centers for Disease Control and Prevention, 2013 *DENV-1–4 Real-Time RT-PCR Assay for Detection and Serotype Identification of Dengue Virus* Atlanta, GA: Centers for Disease Control and Prevention. Available at: https://www.cdc.gov/dengue/resources/rt-pcr/cdcpackageinsert.pdf. Accessed December 6, 2017.

[40] Santiago GA Vergne E Quiles Y Cosme J Vazquez J Medina JF Medina F Colón C Margolis H Muñoz-Jordán JL, 2013 Analytical and clinical performance of the CDC real time RT-PCR assay for detection and typing of dengue virus. PLoS Negl Trop Dis 7: e2311. 23875046 10.1371/journal.pntd.0002311 PMC3708876

[41] Lanciotti RS Kosoy OL Laven JJ Velez JO Lambert AJ Johnson AJ Stanfield SM Duffy MR, 2008 Genetic and serologic properties of Zika virus associated with an epidemic, Yap State, Micronesia, 2007. Emerg Infect Dis 14: 1232–1239. 18680646 10.3201/eid1408.080287 PMC2600394

[42] Bolger AM Lohse M Usadel B, 2014 Trimmomatic: a flexible trimmer for Illumina sequence data. Bioinformatics 30: 2114–2120. 24695404 10.1093/bioinformatics/btu170 PMC4103590

[43] Li H, 2013 *Aligning Sequence Reads, Clone Sequences and Assembly Contigs with BWA-MEM.* ArXiv Prepr ArXiv13033997. Available at: http://arxiv.org/abs/1303.3997. Accessed November 11, 2016.

[44] Li H Handsaker B Wysoker A Fennell T Ruan J Homer N Marth G Abecasis G Durbin R, 2009 1000 genone project data processing subgroup. The sequence alignment/map format and SAMtools. Bioinformatics 25: 2078–2079. 19505943 10.1093/bioinformatics/btp352 PMC2723002

[45] Hunter JD, 2007 Matplotlib: a 2D graphics environment. Comput Sci Eng 9: 90–95.

[46] Tamura K Stecher G Peterson D Filipski A Kumar S, 2013 MEGA6: molecular evolutionary genetics analysis version 6.0. Mol Biol Evol 30: 2725–2729. 24132122 10.1093/molbev/mst197 PMC3840312

[47] Altschul SF Gish W Miller W Myers EW Lipman DJ, 1990 Basic local alignment search tool. J Mol Biol 215: 403–410. 2231712 10.1016/S0022-2836(05)80360-2

[48] Posada D, 2008 jModelTest: phylogenetic model averaging. Mol Biol Evol 25: 1253–1256. 18397919 10.1093/molbev/msn083

[49] Guindon S Gascuel O, 2003 A simple, fast, and accurate algorithm to estimate large phylogenies by maximum likelihood. Syst Biol 52: 696–704. 14530136 10.1080/10635150390235520

[50] Guindon S Dufayard J-F Lefort V Anisimova M Hordijk W Gascuel O, 2010 New algorithms and methods to estimate maximum-likelihood phylogenies: assessing the performance of PhyML 3.0. Syst Biol 59: 307–321. 20525638 10.1093/sysbio/syq010

[51] Pyke AT Moore PR Taylor CT Hall-Mendelin S Cameron JN Hewitson GR Pukallus DS Huang B Warrilow D van den Hurk AF, 2016 Highly divergent dengue virus type 1 genotype sets a new distance record. Sci Rep 6: 22356. 26924208 10.1038/srep22356 PMC4770315

[52] Fry SR 2011 The diagnostic sensitivity of dengue rapid test assays is significantly enhanced by using a combined antigen and antibody testing approach. PLoS Negl Trop Dis 5: e1199. 21713023 10.1371/journal.pntd.0001199 PMC3119643

[53] Beckett CG 2005 Early detection of dengue infections using cluster sampling around index cases. Am J Trop Med Hyg 72: 777–782. 15967759

[54] Reiner RC Jr 2014 Time-varying, serotype-specific force of infection of dengue virus. Proc Natl Acad Sci USA 111: E2694–E2702. 24847073 10.1073/pnas.1314933111 PMC4084484

[55] Rico-Hesse R, 2010 Dengue virus virulence and transmission determinants. *Dengue Virus* New York, NY: Springer, 45–55. 10.1007/978-3-642-02215-9_4 PMC3057078 19802577

[56] Junxiong P Yee-Sin L, 2015 Clustering, climate and dengue transmission. Expert Rev Anti Infect Ther 13: 731–740. 25872683 10.1586/14787210.2015.1028364

[57] Standish K Kuan G Avilés W Balmaseda A Harris E, 2010 High dengue case capture rate in four years of a cohort study in Nicaragua compared to national surveillance data. PLoS Negl Trop Dis 4: e633. 20300515 10.1371/journal.pntd.0000633 PMC2838781

[58] Lustig Y Mendelson E Paran N Melamed S Schwartz E, 2016 Detection of Zika virus RNA in whole blood of imported Zika virus disease cases up to 2 months after symptom onset, Israel, December 2015 to April 2016. Euro Surveill 21 Available at: http://www.e-sciencecentral.org/articles/SC000017361. Accessed May 11, 2017. 10.2807/1560-7917.ES.2016.21.26.30269 27386894

[59] Gourinat A-C O’Connor O Calvez E Goarant C Dupont-Rouzeyrol M, 2015 Detection of Zika virus in urine. Emerg Infect Dis 21: 84–86. 25530324 10.3201/eid2101.140894 PMC4285245

[60] Ali A 2013 Seroepidemiology of dengue fever in Khyber Pakhtunkhawa, Pakistan. Int J Infect Dis 17: e518–e523. 23523057 10.1016/j.ijid.2013.01.007

[61] Fernández E Smieja M Walter SD Loeb M, 2016 A predictive model to differentiate dengue from other febrile illness. BMC Infect Dis 16: 694. 27876005 10.1186/s12879-016-2024-y PMC5120437

[62] Zim MM Sam I-C Omar SS Chan YF AbuBakar S Kamarulzaman A, 2013 Chikungunya infection in Malaysia: comparison with dengue infection in adults and predictors of persistent arthralgia. J Clin Virol 56: 141–145. 23201456 10.1016/j.jcv.2012.10.019

[63] Murray KO 2013 Identification of dengue fever cases in Houston, Texas, with evidence of autochthonous transmission between 2003 and 2005. Vector Borne Zoonotic Dis 13: 835–845. 24107180 10.1089/vbz.2013.1413 PMC3868290

[64] Parreira R 2014 Angola’s 2013 dengue outbreak: clinical, laboratory and molecular analyses of cases from four Portuguese institutions. J Infect Dev Ctries 8: 1210–1215. 25212088 10.3855/jidc.4910

[65] Thai KT 2010 Clinical, epidemiological and virological features of dengue virus infections in Vietnamese patients presenting to primary care facilities with acute undifferentiated fever. J Infect 60: 229–237. 20080126 10.1016/j.jinf.2010.01.003 PMC2954363

[66] Waggoner JJ 2016 Viremia and clinical presentation in Nicaraguan patients Infected with Zika virus, chikungunya virus, and dengue virus. Clin Infect Dis 63: 1584–1590. 27578819 10.1093/cid/ciw589 PMC5146717

[67] Thomas L 2008 Influence of the dengue serotype, previous dengue infection, and plasma viral load on clinical presentation and outcome during a dengue-2 and dengue-4 co-epidemic. Am J Trop Med Hyg 78: 990–998. 18541782

[68] Le Gonidec E 2016 Clinical survey of dengue virus circulation in the republic of Djibouti between 2011 and 2014 identifies serotype 3 epidemic and recommends clinical diagnosis guidelines for resource limited settings. PLoS Negl Trop Dis 10: e0004755. 27322644 10.1371/journal.pntd.0004755 PMC4920588

[69] Balmaseda A 2010 Trends in patterns of dengue transmission over 4 years of a pediatric cohort study in Nicaragua. J Infect Dis 201: 5–14. 19929380 10.1086/648592 PMC3724236

[70] Endy TP Anderson KB Nisalak A Yoon I-K Green S Rothman AL Thomas SJ Jarman RG Libraty DH Gibbons RV, 2011 Determinants of inapparent and symptomatic dengue infection in a prospective study of primary school children in Kamphaeng Phet, Thailand. PLoS Negl Trop Dis 5: e975. 21390158 10.1371/journal.pntd.0000975 PMC3046956

[71] Handel AS Ayala EB Borbor-Cordova MJ Fessler AG Finkelstein JL Espinoza RXR Ryan SJ Stewart-Ibarra AM, 2016 Knowledge, attitudes, and practices regarding dengue infection among public sector healthcare providers in Machala, Ecuador. Trop Dis Travel Med Vaccines 2: 8. 28883952 10.1186/s40794-016-0024-y PMC5531027

[72] Lowe R Stewart-Ibarra AM Petrova D García-Díez M Borbor-Cordova MJ Mejía R Regato M Rodó X, 2017 Climate services for health: predicting the evolution of the 2016 dengue season in Machala, Ecuador. Lancet Planet Health 1: e142–e151. 10.1016/S2542-5196(17)30064-5 29851600

[73] Viennet E Harley D, 2017 Climate services for health: cooperation for climate informed dengue surveillance. Lancet Planet Health 1: e126–e127. 10.1016/S2542-5196(17)30065-7 29851596

[74] Shepard DS Coudeville L Halasa YA Zambrano B Dayan GH, 2011 Economic impact of dengue illness in the Americas. Am J Trop Med Hyg 84: 200–207. 21292885 10.4269/ajtmh.2011.10-0503 PMC3029168

[75] Heydari N Larsen DA Neira M Beltrán Ayala E Fernandez P Adrian J Rochford R Stewart-Ibarra AM, 2017 Household dengue prevention interventions, expenditures, and barriers to *Aedes aegypti* control in Machala, Ecuador. Int J Environ Res Public Health 14: 196. 10.3390/ijerph14020196 PMC5334750 28212349

[76] Krisher LK 2016 Successful malaria elimination in the Ecuador–Peru border region: epidemiology and lessons learned. Malar J 15: 573. 27894320 10.1186/s12936-016-1630-x PMC5126842

[77] Ponce P Morales D Argoti A Cevallos VE, 2018 First report of *Aedes (Stegomyia) albopictus* (Skuse) (Diptera: Culicidae), the Asian Tiger Mosquito, in Ecuador. J Med Ento 55: 248–249. 10.1093/jme/tjx165 PMC5850216 29029173

