# Supplementary material for: The Burden of Dengue Fever and Chikungunya in Southern Coastal Ecuador: Epidemiology, Clinical Presentation, and Phylogenetics from the First Two Years of a Prospective Study
Source: Am J Trop Med Hyg. 2018 Mar 5;98(5):1444–59. doi: 10.4269/ajtmh.17-0762 (PMC5953373; doi:10.4269/ajtmh.17-0762)
Supplement: Supplementary file 1 [file tpmd170762.SD1.pdf]

Supplemental Table 1

The prevalence of dengue-like symptoms in associates with acute DENV infections

Symptoms	N = 50	Prevalence (%)
Any dengue-like symptom	34	68
Temperature > 38°C	2	4
Symptoms in prior 7 days		
Headache	16	32
Drowsiness/lethargy	12	24
Fever	11	22
Muscle/joint pain	11	22
Retro-orbital pain	11	22
Abdominal pain	9	18
Rash	9	18
Anorexia and nausea	5	10
Diarrhea	3	6
Vomiting	2	4
Bleeding	1	2

DENV = dengue virus. Dengue-like symptoms include all symptoms listed in the table. Symptoms are presented from most to least prevalent.

Supplemental Table 2

Primers used for reverse transcription polymerase chain reaction (RT-PCR) diagnosis of DENV, CHIKV, and Zika virus

Viral target	Primer name	Primer sequence 5′ to 3′
DENV1	D1F	CAAAAGGAAGTCGYGCAATA
DENV1	D1R	CTGAGTGAATTCTCTCTGCTRAAC
DENV2	D2F	CAGGCTATGGCACYGTCACGAT
DENV2	D2R	CCATYTGCAGCARCACCATCTC
DENV3	D3F	GGACTRGACACACGCACCCA
DENV3	D3R	CATGTCTCTACCTTCTCGACTTGYCT
DENV4	D4F	TTGTCCTAATGATGCTRGTCG
DENV4	D4R	TCCACCYGAGACTCCTTCCA
CHIKV	CHIKF_856	ACCATCGGTGTTCCATCTAAAG
CHIKV	CHIKR_962c	GCCTGGGCTCATCGTTATT
ZIKV	ZIKAF_1086	CCGCTGCCCAACACAAG
ZIKV	ZIKAR_1162c	CCACTAACGTTC TTTTGCAGACAT

CHIKV = chikungunya virus; DENV = dengue virus.

Supplemental Table 3

Probes used for reverse transcription polymerase chain reaction (RT-PCR) diagnostics of DENV, CHIKV, and ZIKV

Viral target	Probe name	Probe sequence 5′ to 3′	5′ label	3′ quench
DENV1	D1P	CATGTGGYTGGGAGCRCGC	FAM	BHQ1
DENV2	D2P	CTCYCCRAGAACGGGCCTCGACTTCAA	HEX	BHQ1
DENV3	D3P	ACCTGGATGTCGGCTGAAGGAGCTTG	TexRed	BHQ2
DENV4	D4P	TYCCTACYCCTACGCATCGCATTCCG	Cy5	BHQ3
CHIKV	CHIKP_908	ACAGTGGTT/ZEN/TCGTGTGAGGGCTAC	HEX	IBFQ
ZIKV	ZIKAP_1107	AGCCTACCT/ZEN/TGACAAGCAGTCAGACACTCAA	FAM	IBFQ

CHIKV = chikungunya virus; DENV = dengue virus; ZIKV = Zika virus.

Supplemental Table 4

DENV serotypes

	2014		2015	
	Index cases	Associates	Index cases	Associates
DENV serotypes	N = 51	N = 18	N = 23	N = 1
1	4/51 (7.8%)	3/18 (16.7%)	14/23 (60.9%)	0/1 (0%)
1 and 2	1/51 (2.0%)	0/18 (0%)	0/23 (0%)	0/1 (0%)
2	43/51 (84.3%)	10/18 (55.6%)	9/23 (39.1%)	1/1 (100%)
3	2/51 (3.9%)	5/18 (27.8%)	0/23 (0%)	0/1 (0%)
4	1/51 (2.0%)	0/18 (0%)	0/23 (0%)	0/1 (0%)

DENV = dengue virus. Results from the analysis of samples from 69 individuals in 2014 and 24 individuals in 2015 that were serotyped for DENV by reverse transcription polymerase chain reaction. In 2014, all four DENV serotypes were detected, with DENV2 as the predominant serotype. One index case in 2014 was positive for DENV1 and DENV2. In 2015, DENV1 and DENV2 co-circulated and DENV1 was the predominant serotype.

Supplemental Table 5

DENV serology results for index cases and associates

Serology	2014		2015	
	Index cases	Associates	Index cases	Associates
	N = 99	*N* = 81	*N* = 31	*N* = 6
Primary DENV infection	26 (26.3%)	38 (46.9%)	21 (67.7%)*	4 (66.7%)*
Secondary DENV infection	73 (73.7%)	43 (53.0%)	10 (32.2%)†	2 (33.3%)

CHIKV = chikungunya virus; DENV = dengue virus. The prevalence of primary and secondary DENV infections as a proportion of individuals who had an acute or recent DENV infection and had valid serology results (217/284 individuals with acute or recent DENV infections, as reported in Table 1). Secondary DENV infections were more prevalent in 2014, whereas primary DENV infections were more prevalent in 2015. The serology of index cases in 2014 vs. 2015 was significantly different ($P < 0.001$). The serology of associates in 2014 vs. 2015 was not significantly different ($P > 0.05$).

* Includes four index cases and one associate with acute CHIKV infections.

† Includes one index case with acute CHIKV infections.

Supplemental Table 6

Characteristics of acute DENV infections

Characteristics	2014		2015	
	Index cases	Associates	Index cases	Associates
	N = 75	*N* = 45	*N* = 24	*N* = 5
Age in years, mean (SD)	20.7 (15.7)	25.2 (18.6)	19.3 (12.8)	19.6 (14.6)
Gender, % female	28/75 (37.3%)	29/45 (64.4%)	13/24 (54.1%)	2/4 (50.0%)
Temperature > 38°C	16/75 (21.3%)	2/43 (4.7%)	10/24 (41.7%)	0/5 (0%)
Fever in the last 7 days	73/75 (97.3%)	10/41 (24.4%)	24/24 (100%)	1/5 (20.0%)
Hospitalized	12/75 (16.0%)	Not applicable	8/24 (33.3%)	Not applicable

DENV = dengue virus; SD = standard deviation. Index cases and associates with acute DENV infections in 2014 and 2015: mean age (SD) and gender, febrile status, and the proportion who were hospitalized. There were no significant differences between years ($P > 0.05$).

Supplemental Table 7

The prevalence of symptomatic acute (SA) DENV and CHIKV infections by age classes

Age class	Index cases DENV (2014, 2015)			Associates DENV (2014, 2015)			Index cases CHIKV (2015)		
	SA	*N*	Prevalence (%)	SA	*N*	Prevalence (%)	SA	*N*	Prevalence (%)
0–9	23	65	35.4	5	22	22.7	4	18	22.2
10–19	40	97	41.2	15	71	21.1	9	31	29.0
20–29	13	51	25.5	4	66	6.1	8	25	32.0
30–39	9	39	23.1	5	59	8.5	9	16	56.3
40–49	6	25	24.0	1	62	1.6	9	14	64.3
50–59	7	21	33.3	1	47	2.1	7	10	70.0
60–79	1	12	8.3	3	54	5.6	7	9	77.8
Total	99	310	31.9	34	381*	8.9	53	123	43.1

CHIKV = chikungunya virus; DENV = dengue virus; SA = symptomatic acute. Data were used to generate Figure 4. Index cases and associates with SA DENV or CHIKV infections, as a proportion of all individuals from the age class who were recruited into the study (*N*). For DENV, data are combined for 2014 and 2015. For CHIKV, data are shown only for 2015. There were no associates with SA CHIKV infections.

* Three associates were missing age information.

Supplemental Table 8

The prevalence of primary and secondary DENV infections by age class

Age class	Index cases (*N* = 130)		Associates (*N* = 87)	
	Primary	Secondary	Primary	Secondary
0–9	13/26 (50.0%)	13/26 (50.0%)	4/5 (80.0%)	1/5 (20.0%)
10–19	15/41 (36.6%)	26/41 (63.4%)	12/22 (54.5%)	10/22 (45.5%)
20–29	9/27 (33.3%)	18/27 (66.7%)	7/14 (50.0%)	7/14 (50.0%)
30–39	4/20 (20.0%)	16/20 (80.0%)	8/19 (42.1%)	11/19 (57.9%)
40–49	1/6 (16.7%)	5/6 (83.3%)	6/12 (50.0%)	6/12 (50.0%)
50–59	4/8 (50.0%)	4/8 (50.0%)	4/8 (50.0%)	4/8 (50.0%)
60–79	1/2 (50.0%)	1/2 (50.0%)	1/7 (14.3%)	6/7 (85.7%)
Total	47/130 (36.2%)	83/130 (63.8%)	42/87 (48.3%)	45/87 (51.7%)

DENV = dengue virus. Data were used to generate Figure 4. The proportion of primary and secondary DENV infections per age class for index cases and associates with valid serology and acute or recent DENV infections in 2014 and 2015 combined.

Supplemental Table 9

Demographics and symptoms associated with primary vs. secondary DENV infections in index cases that had acute or recent (AR) DENV infections

	Primary infections	Secondary infections	
	N = 43	*N* = 82	*P* value
Age in years, mean (SD)	18.0 (13.1)	23.2 (13.8)	**0.046**
Gender, % female	19/43 (44.2%)	41/82 (50.0%)	0.53
Temperature > 38°C	10/43 (23.3%)	7/81 (8.6%)	**0.048**
Hospitalized	4/43 (9.3%)	31/82 (37.8%)	**0.002**
Symptoms in prior 7 days			
Fever	42/43 (97.7%)	77/81 (95.1%)	0.66
Headache	37/43 (86.0%)	62/82 (75.6%)	0.17
Anorexia and nausea	27/143 (62.8%)	53/82 (64.6%)	0.84
Muscle/joint pain	33/43 (76.7%)	62/82 (75.6%)	0.89
Rash	9/42 (21.4%)	16/82 (19.5%)	0.80
Bleeding	3/42 (7.4%)	12/82 (14.6%)	0.26
Vomiting	15/43 (34.9%)	45/82 (54.9%)	**0.03**
Drowsiness/lethargy	36/43 (83.7%)	74/82 (90.2%)	0.29
Abdominal pain	25/42 (59.5%)	53/82 (64.6%)	0.58
Diarrhea	10/43 (23.3%)	25/82 (30.5%)	0.39
Retro-orbital pain	32/43 (74.4%)	48/81 (59.3%)	0.09

DENV = dengue virus; SD = standard deviation. Index cases with secondary DENV infections were significantly older, were less likely to have a fever, and were more likely to report vomiting ($P < 0.05$). Hospitalized cases were more likely to have secondary infections. Index cases with DENV and chikungunya virus coinfections were excluded (four primary infections and one secondary infection). Bolded text denotes statistical significance ($P < 0.05$).

Supplemental Table 10

Demographics and symptoms associated with DENV1 vs. DENV2 infections in index cases

	DENV1	DENV2	
	N = 18	*N* = 51	*P* value
Age in years, mean (SD)	14.7 (10.5)	25.2 (16.2)	**0.01**
Gender, % female	9/18 (50.0%)	21/51 (41.2%)	0.71
Temperature > 38°C	8/18 (44.4%)	15/51 (29.4%)	0.38
Hospitalized	5/18 (27.8%)	7/51 (13.7%)	0.32
Symptoms in prior 7 days			
Fever	18/18 (100%)	49/51 (96.1%)	0.97
Headache	17/18 (94.4%)	43/51 (84.3%)	0.49
Anorexia and nausea	14/18 (77.8%)	32/51 (62.8%)	0.38
Muscle/joint pain	12/18 (66.7%)	43/51 (84.3%)	0.21
Rash	2/17 (11.8%)	8/51 (15.7%)	1.00
Bleeding	3/18 (16.7%)	2/51 (3.92%)	0.21
Vomiting	9/18 (50.0%)	26/51 (51.0%)	1.00
Drowsiness/lethargy	16/18 (88.9%)	44/51 (86.3%)	1.00
Abdominal pain	13/18 (72.2%)	31/51 (60.8%)	0.56
Diarrhea	4/18 (22.2%)	12/51 (23.5%)	1.00
Retro-orbital pain	13/18 (72.2%)	36/51 (70.6%)	1.00

DENV = dengue virus; SD = standard deviation. Index cases with DENV1 infections were significantly younger than those with DENV2 infections ($P < 0.05$). For all other measures, there were no significant differences ($P > 0.05$). One index case with a DENV and chikungunya virus coinfection was excluded. Bolded text denotes statistical significance ($P < 0.05$).

Supplemental Table 11

DENV infections per cluster

Year	Cluster	SAR	AR	*N* (initiate index + associates)
2014	1	2	3	8
	2	1	1	7
	3	3	4	12
	4	2	2	15
	5	1	2	8
	6	4	5	10
	7	3	6	12
	8	7	7	13
	9	5	5	10
	10	2	2	7
	11	3	3	11
	12	3	3	8
	13	1	5	9
	14	4	4	11
	15	4	5	9
	16	6	6	11
	17	5	8	15
	18	5	6	10
	19	5	5	9
	20	3	6	12
	21	5	10	18
	22	7	8	9
	23	5	8	13
	24	3	4	12
	25	2	2	8
	26	4	4	13
	27	2	3	6
	28	2	2	11
	29	2	4	9
	30	1	1	7
	31	2	3	8
	32	1	1	9
	Mean (SD)	3.3 (1.7)	4.3 (2.3)	10.3 (2.7)
2015	1	1	2	10
	2	1	2	10
	3	1	2	8
	4	1	1	5
	5	1	1	8
	6	3	3	8
	7	1	2	13
	8	1	1	6
	9	2	3	8
	10	2	2	6
	11	2	2	9
	12	1	2	7
	Mean (SD)	1.4 (0.7)	1.9 (0.7)	8.2 (2.2)
Overall 2014 and 2015	Mean (SD)	2.8 (1.7)	3.7 (2.3)	9.7 (2.7)

DENV = dengue virus; SA = symptomatic acute; SD = standard deviation. The numbers of symptomatic acute or recent (SAR) DENV infections and acute or recent (AR) DENV infections per cluster, and the total number of people per cluster. Each cluster includes one initiate index case, which by definition was an SAR infection. Means and SD for clusters are shown for each year and for both years combined. All measures were significantly greater in 2014 than in 2015 ($P < 0.05$).
